# Development and Replication of Objective Measurements of Social Visual Engagement to Aid in Early Diagnosis and Assessment of Autism

**DOI:** 10.1001/jamanetworkopen.2023.30145

**Published:** 2023-09-05

**Authors:** Warren Jones, Cheryl Klaiman, Shana Richardson, Meena Lambha, Morganne Reid, Taralee Hamner, Chloe Beacham, Peter Lewis, Jose Paredes, Laura Edwards, Natasha Marrus, John N. Constantino, Sarah Shultz, Ami Klin

**Affiliations:** 1 Marcus Autism Center, Children’s Healthcare of Atlanta, Atlanta, Georgia; 2 Division of Autism and Related Disabilities, Department of Pediatrics, Emory University School of Medicine, Atlanta, Georgia; 3 Center for Translational Social Neuroscience, Emory University, Atlanta, Georgia; 4 Department of Psychiatry, Washington University in St Louis School of Medicine, St Louis, Missouri; 5 Intellectual and Developmental Disabilities Research Center, Washington University in St Louis School of Medicine, St Louis, Missouri; 6 Now with Department of Psychiatry, Emory University School of Medicine, Atlanta, Georgia; 7 Now with Division of Behavioral and Mental Health, Children’s Healthcare of Atlanta, Atlanta, Georgia

## Abstract

**Importance:**

Autism spectrum disorder is a common and early-emerging neurodevelopmental condition. While 80% of parents report having had concerns for their child’s development before age 2 years, many children are not diagnosed until ages 4 to 5 years or later.

**Objective:**

To develop an objective performance-based tool to aid in early diagnosis and assessment of autism in children younger than 3 years.

**Design, Setting, and Participants:**

In 2 prospective, consecutively enrolled, broad-spectrum, double-blind studies, we developed an objective eye-tracking–based index test for children aged 16 to 30 months, compared its performance with best-practice reference standard diagnosis of autism (discovery study), and then replicated findings in an independent sample (replication study). Discovery and replication studies were conducted in specialty centers for autism diagnosis and treatment. Reference standard diagnoses were made using best-practice standardized protocols by specialists blind to eye-tracking results. Eye-tracking tests were administered by staff blind to clinical results. Children were enrolled from April 27, 2013, until September 26, 2017. Data were analyzed from March 28, 2018, to January 3, 2019.

**Main Outcomes and Measures:**

Prespecified primary end points were the sensitivity and specificity of the eye-tracking–based index test compared with the reference standard. Prespecified secondary end points measured convergent validity between eye-tracking–based indices and reference standard assessments of social disability, verbal ability, and nonverbal ability.

**Results:**

Data were collected from 1089 children: 719 children (mean [SD] age, 22.4 [3.6] months) in the discovery study, and 370 children (mean [SD] age, 25.4 [6.0] months) in the replication study. In discovery, 224 (31.2%) were female and 495 (68.8%) male; in replication, 120 (32.4%) were female and 250 (67.6%) male. Based on reference standard expert clinical diagnosis, there were 386 participants (53.7%) with nonautism diagnoses and 333 (46.3%) with autism diagnoses in discovery, and 184 participants (49.7%) with nonautism diagnoses and 186 (50.3%) with autism diagnoses in replication. In the discovery study, the area under the receiver operating characteristic curve was 0.90 (95% CI, 0.88-0.92), sensitivity was 81.9% (95% CI, 77.3%-85.7%), and specificity was 89.9% (95% CI, 86.4%-92.5%). In the replication study, the area under the receiver operating characteristic curve was 0.89 (95% CI, 0.86-0.93), sensitivity was 80.6% (95% CI, 74.1%-85.7%), and specificity was 82.3% (95% CI, 76.1%-87.2%). Eye-tracking test results correlated with expert clinical assessments of children’s individual levels of ability, explaining 68.6% (95% CI, 58.3%-78.6%), 63.4% (95% CI, 47.9%-79.2%), and 49.0% (95% CI, 33.8%-65.4%) of variance in reference standard assessments of social disability, verbal ability, and nonverbal cognitive ability, respectively.

**Conclusions and Relevance:**

In two diagnostic studies of children younger than 3 years, objective eye-tracking–based measurements of social visual engagement quantified diagnostic status as well as individual levels of social disability, verbal ability, and nonverbal ability in autism. These findings suggest that objective measurements of social visual engagement can be used to aid in autism diagnosis and assessment.

## Key Points

**Question:**

Can objective measurements of social visual engagement be developed and replicated to aid in early diagnosis and assessment of autism before age 3 years?

**Findings:**

In 2 prospective double-blind studies of diagnostic performance in 1089 children aged 16 to 30 months, 719 in discovery and 370 in replication, eye-tracking–based measurements of social visual engagement relative to expert clinical diagnosis had area under the receiver operating characteristic curve of 0.90, sensitivity of 81.9%, and specificity of 89.9% in discovery; and area under the curve of 0.89, sensitivity of 80.6%, and specificity of 82.3% in replication.

**Meaning:**

These results offer the prospect of an objective biomarker to aid in autism diagnosis and assessment.

## Teaser

This diagnostic study develops an objective performance-based tool and assesses its ability to aid in the early diagnosis and assessment of autism in children younger than 3 years.

## Introduction

Approximately 1 in 36 US children is affected by autism.^1^ Thirty percent of parents of children with autism had concerns for their child’s development before age 12 months, 50% of parents had concerns by age 18 months, and 80% had concerns by age 2 years.^2,3,4^ Despite these early concerns and the manifest behaviors that elicited these concerns, the median age of US diagnosis remains delayed until the age of 4 to 5 years.^5,6^ The age of diagnosis is even later among those who lack resources or lack access to expert clinicians: diagnoses for US racial minority families, families with low income, and families residing in rural areas lag further.^1,6,7,8,9^

The goal of diagnosis in autism is to facilitate timely and targeted support to help a child and family as needed. To that end, there may be an important role for new tools and objective biomarkers that can accurately and efficiently aid in diagnosing children as well as aid in quantifying individual strengths and vulnerabilities.^10^ Such tools could enhance health care system capacity and help facilitate timely access to individually appropriate services.^10,11^

In current best practice, autism is diagnosed behaviorally by symptomatic deficits in social interaction and communication and by the presence of restricted and repetitive behaviors.^12^ Current gold (reference) standard^13^ diagnostic instruments are standardized validated assessments that measure the presence of autistic social disability through both behavioral observation and parent interview.^14,15^ Best-practice guidelines also call for standardized assessments of a child’s cognitive and language skills.^16^

Unfortunately, there are often long wait lists to access expert clinicians using gold standard instruments (a situation now described as a crisis)^17^ and community use of gold standard instruments is limited.^18,19^ Consequently, many children experience delayed diagnosis, and most receive diagnostic labels without receiving comprehensive evaluations and standardized assessments.^18,19^

In the present studies, we tested the performance of eye-tracking–based measurements of social visual engagement to accurately predict autism diagnoses and to objectively quantify individual levels of social disability, verbal ability, and nonverbal cognitive ability. In primary analyses, we measured the sensitivity and specificity of eye-tracking assays in comparison with clinician best-estimate diagnosis by expert clinicians. In secondary analyses, we quantified convergent validity between eye-tracking–based indices of social disability, verbal ability, and nonverbal cognitive ability in comparison with standardized assessments thereof as administered by expert clinicians.^20,21^

The present studies build on prior research using eye tracking to quantify social visual engagement, defined as how children look at and learn from their surrounding social environment. The prior research found that social visual engagement is strongly influenced by individual genetic variation (with monozygotic twin-twin concordance of approximately 0.9),^22^ is highly biologically conserved,^23^ and is atypical in autism^24,25^ from very early ages in development (ie, 2-6 months).^26^ Here we test the hypothesis that measurements of social visual engagement collected via eye tracking can serve as a robust biomarker to enable early diagnosis and assessment of autism.

## Methods

The goal of the current studies was to evaluate performance of eye-tracking–based assays to accurately assess categorical presence of autism and to measure dimensional levels of ability or disability. In design, terminology, and reporting, this research followed the Standards for Reporting of Diagnostic Accuracy (STARD) guidelines,^27,28,29^ with eye-tracking assays referred to as the “index test,” and expert clinical diagnosis using standardized assessments referred to as the “reference standard.” Two observational studies were conducted: a discovery study that was used to develop the data collection tool and algorithms, and a replication study that was used to test performance in an independent sample. Children were consecutively enrolled from April 27, 2013, until September 26, 2017. Data were analyzed from March 28, 2018, to January 3, 2019. The research protocol was approved by the institutional review boards of Emory University and Washington University in St Louis. Written informed consent was obtained from all parents or legal guardians.

### Study Design

To eliminate or minimize design-related bias (as highlighted by Lijmer et al^30^), data were collected prospectively; participants were enrolled consecutively; enrolled participants had a broad spectrum of case presentation (spanning the full spectrum of symptom severity and absence of symptoms); clinical assessments were blind to eye tracking, eye tracking was blind to clinical assessments; and the index test and reference standard diagnosis were performed with all participants. Only the results of best-practice standardized assessments and reference standard clinician best-estimate diagnosis^31^ were used clinically or communicated to parents. In this way, best-practice standard of care was maintained for all participants, blind to eye-tracking results; neither a child’s parents nor expert clinical staff were informed of a child’s eye-tracking results.

### Participants

**Figure 1.**
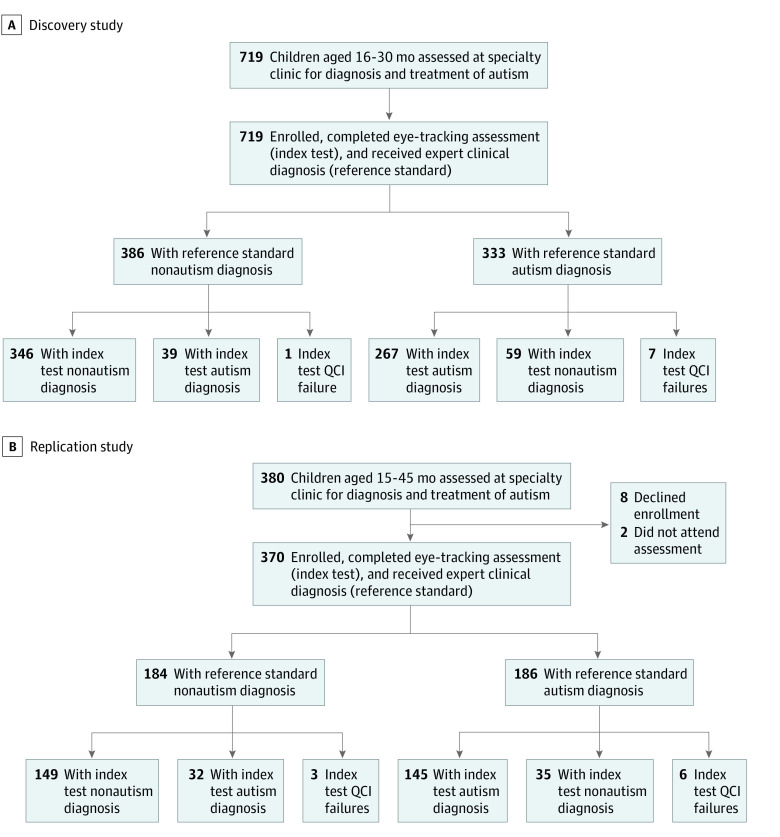
Participant Enrollment and Outcomes for Comparing Objective Measurements of Social Visual Engagement With Expert Clinical Diagnosis of Autism in Discovery and Replication Studies A, Participant flow for the discovery study. B, Participant flow for the replication study. In both studies, during a single visit at the testing site, enrolled participants received expert clinical diagnosis using standardized assessments (reference standard diagnosis) as well as eye-tracking–based measurement of social visual engagement (index test). Index test quality control indicator (QCIs) failures occurred when participants’ data failed to meet automated preset data QCIs (additional details are available in eTables 2 and 3 in Supplement 1).

A total of 1089 children participated: 719 participated in the discovery study, and 370 participated in the replication study (Figure 1). Eligible participants were identified on the basis of chronological age and were recruited through placement of advertising materials in local media, specialty clinics, and pediatric practices. The studies were designed to develop and test a tool to aid in the diagnosis and assessment of autism, not to test the tool’s utility as a screening instrument. To that end, children for whom there were concerns about autism were recruited (ie, children typically evaluated in specialized clinics for the diagnosis of autism). To be eligible for participation, children could not have clinically meaningful hearing or visual impairments (eg, congenital deafness, blindness, or nystagmus); could not have previously diagnosed genetic conditions associated with autism-related symptoms (eg, not known to have fragile X or Rett syndromes); had to be generally healthy at the time of testing, with no acute illness; had to be either born at or after 37 weeks’ gestational age (discovery study) or born at or after 32 weeks’ gestational age (replication study); and had to be either between the ages of 16 and 30 months (discovery study) or between the ages of 16 and 45 months (replication study). In both studies, the age of participants was guided by future intended use (ie, to align in time with ages that would ideally enable diagnosed children to be referred to early intervention before age 36 months). In the replication study, to test performance among a broader range of children, increased prematurity at birth and older age at enrollment was allowed. For the purpose of sample characterization, patient demographic data (including race, ethnicity, and maternal educational level) were collected by parents’ selection of fixed categories. Race and ethnicity data were collected to enable evaluation of whether test performance varied based on these characteristics. Further details are available in the eMethods in Supplement 1.

### Reference Standard Diagnosis

Reference standard diagnosis consisted of clinician best-estimate diagnosis^32,33,34,35^ by experienced licensed clinicians using standardized diagnostic protocols and developmental assessments.^20^ Reference standard diagnosis was assigned based on all available clinical information, including developmental assessments as well as medical and developmental history gathered in clinical interviews. At young ages, clinician best-estimate diagnosis (ie, experienced clinicians’ judgments using the totality of information available) is a more stable predictor of later diagnosis than strict reliance on cutoff scores.^20,36^ For example, while scores on the Autism Diagnostic Observation Schedule, second edition (ADOS-2), may vary during the first 2 to 3 years of life, clinician best estimate is more stable.^32,36^ The standardized diagnostic protocol was sequential so that a child’s developmental history and scores on screening tests dictated subsequent assessments. A complete description of this protocol is available in the Reference Standard Diagnostic Assessment Procedures subsection of the eMethods in Supplement 1.

### Index Test Eye Tracking

For index test measurements of social visual engagement, eye-tracking data were collected while participants watched video scenes of social interaction (examples are provided in eFigure 1 in Supplement 1). Fourteen video scenes were presented, each with a mean (SD) duration of approximately 54.0 (21.5) seconds (range, 21.7 seconds to 1 minute, 29.7 seconds; sum, 12 minutes, 35.5 seconds). Experimental procedures, data collection, and data processing were performed as described in prior studies^22^ and in the Experimental Procedures and Data Collection subsection of the eMethods in Supplement 1. Data collection for the discovery study was performed in an academic medical center laboratory setting. Data collection for the replication study was performed in both an academic medical center laboratory setting and a community clinic using a standalone investigational eye-tracking device (eFigure 2 in Supplement 1). Eye-tracking data were collected using near-infrared video-based measurements of eye movements using specialized cameras and hardware (additional details are provided in the Experimental Procedures and Data Collection subsection of the eMethods in Supplement 1).

All collected data underwent automated quality control analyses measuring calibration accuracy, integrity of eye movements, duration of data collected, and time spent fixating on video scenes. Data that met or exceeded predefined automated static quality control thresholds proceeded to analysis (Quality Control Indicators subsection of the eMethods in Supplement 1). All steps in data processing and analysis were automated, with no manual human review or analysis required.

Time-varying kernel density estimation was used to quantify social visual engagement^37^ (eFigure 3 in Supplement 1). Probability density functions of visual fixation and scanning were calculated during each moment of collected eye-tracking data (eFigure 4 in Supplement 1). Moments in time when the majority of participants with nonautism diagnoses in the discovery study fixated on approximately the same location(s) at the same moments at levels greater than expected by chance were identified by permutation testing.^38^ Discovery study data were then mined to identify time points when the majority of participants with autism fixated on alternate locations (defining a classification index). Data were also mined to identify time points when autism and nonautism discovery study data were correlated with measurements of (1) social disability (correlated with ADOS-2 total scores), (2) verbal ability (correlated with verbal age-equivalent scores from the Mullen Scales of Early Learning, hereinafter, Mullen), or (3) nonverbal cognitive ability (correlated with visual reception age-equivalent scores from the Mullen). Data mining for these associations thereby defined 3 indices of individual variability in levels of disability and ability. Further details are provided in the Data Processing subsection of the eMethods in Supplement 1. Discovery study results were tested by leave-one-out cross validation,^39^ with each participant tested as an independent comparison relative to the rest of the sample. All parameters were fixed and then tested again in the independent replication study.

### Statistical Analysis

Primary effectiveness analyses were planned as a comparison between the eye-tracking index test results and the reference standard diagnosis results (either autism or nonautism). Sensitivity and specificity were calculated according to standard practice: sensitivity was calculated as the proportion of participants with reference standard autism diagnoses who had eye-tracking results that also indicated autism; specificity was calculated as the proportion of participants with reference standard nonautism diagnoses who had eye-tracking results that also indicated nonautism. The test positivity threshold was derived in the discovery study using the Youden index^40^; the threshold was then fixed for testing in the independent replication study. Receiver operating characteristic curves, area under the curve, positive predictive value (PPV), negative predictive value (NPV), and accuracy of the eye-tracking index test were also calculated, all with 95% CIs.^41^ Primary end point analyses were tested at a 1-sided significance level of α = .025.

Secondary effectiveness analyses were planned as measurements of correlation between eye-tracking–based severity indices and their respective expert clinician–administered reference standard assessments, including the ADOS-2 total score for social disability, the mean of Mullen receptive and expressive language age-equivalent scores for verbal ability, and the Mullen visual reception age-equivalent score for nonverbal ability. For social disability, the correlation was expected to be negative because higher scores on the ADOS-2 denote greater social disability, whereas for the eye-tracking test, lower scores denote greater social disability. Deming regression^42,43^ was used to quantify the relationships between eye-tracking–based indices and their respective reference standards. Standard regression diagnostics (including Cook distance and difference-in-fits),^44,45^ Pearson correlation coefficients, and adjusted *R*^2^ coefficients^46,47,48^ together with 95% CIs were calculated. Secondary outcome analyses were tested at a 1-sided significance level of α = .025. Data analyses were performed in Matlab R2016a (Mathworks, Inc).

## Results

### Participants

A total of 719 children (mean [SD] age, 22.4 [3.6] months; 224 [31.2%] female and 495 [68.8%] male) were enrolled in the discovery study. A total of 370 children (mean [SD] age, 25.4 [6.0] months; 120 [32.4%] female and 250 [67.6%] male) were enrolled in the replication study. Based on reference standard diagnosis, the discovery study comprised 386 participants (53.7%) with nonautism diagnoses and 333 (46.3%) with autism diagnoses, while the replication study comprised 184 participants (49.7%) with nonautism diagnoses and 186 (50.3%) with autism diagnoses.

Participant characteristics and demographic data are shown in the Table. In both studies, participants with autism had higher ADOS-2 domain and total scores (all *t* > 24.0, all *P* < .001). Participants with autism also had lower Mullen verbal age-equivalent scores (both *t* > 7.4, *P* < .001) and lower Mullen nonverbal age-equivalent scores (both *t* > 5.1, *P* < .001). The ADOS-2 scores in both studies indicated that participants with autism represented the full spectrum of autism symptom severity. Likewise, the Mullen scores in both studies indicated that participants with nonautism and autism diagnoses represented a broad range of verbal and nonverbal abilities, extending from substantially delayed to age-appropriate to advanced abilities. In each study, mean (SD) age of the sample with autism diagnoses was significantly older than the sample with nonautism diagnoses (discovery: 23.1 [3.7] months vs 21.7 [3.4] months; *t* = 5.2, *P* <.001; replication: 28.1 [5.8] months vs 22.7 [4.9] months; *t* = 9.7, *P* < .001). Sex differences were as expected,^49^ with a higher number of boys diagnosed with autism in both studies (both *χ^2^* > 16.6; *P* < .001).

**Table.** Participant Characteristics

Reference standard diagnosis	Participants, No. (%)^a^			
	Discovery study (n = 719)		Replication study (n = 370)	
	Nonautism (n = 386)	Autism (n = 333)	Nonautism (n = 184)	Autism (n = 186)
Age, mo				
Mean (SD)	21.7 (3.4)	23.1 (3.7)	22.7 (4.9)	28.1 (5.8)
Median (IQR)	23 (18-24)	24 (20-26)	21 (21-25)	28 (24-31)
Sex				
Female	154 (39.9)	70 (21.0)	78 (42.4)	42 (22.6)
Male	232 (60.1)	263 (79.0)	106 (57.6)	144 (77.4)
Race				
Asian	5 (1.3)	10 (3.0)	1 (0.5)	23 (12.4)
Black or African American	21 (5.4)	67 (20.1)	22 (12.0)	38 (20.4)
Native Hawaiian or Pacific Islander	4 (1.0)	3 (0.9)	2 (1.1)	0
White	281 (72.8)	179 (53.8)	139 (75.6)	106 (57.0)
>1 Race	28 (7.3)	41 (12.3)	19 (10.3)	16 (8.6)
Prefer not to answer or unknown	47 (12.2)	33 (9.9)	1 (0.5)	3 (1.6)
Ethnicity				
Hispanic	24 (6.2)	23 (6.9)	12 (6.5)	20 (10.8)
Non-Hispanic	309 (80.1)	268 (80.5)	166 (90.2)	154 (82.8)
Prefer not to answer or unknown	53 (13.7)	42 (12.6)	6 (3.3)	12 (6.5)
Household income, $				
≤20 000	5 (1.3)	13 (3.9)	14 (7.6)	2 (1.1)
20 001-40 000	17 (4.4)	29 (8.7)	21 (11.4)	16 (8.6)
40 001-60 000	32 (8.3)	48 (14.4)	35 (19.0)	42 (22.6)
60 001-80 000	37 (9.6)	51 (15.3)	31 (16.8)	57 (30.6)
80 001-100 000	51 (13.2)	33 (9.9)	29 (15.8)	29 (15.6)
100 001-125 000	56 (14.5)	26 (7.8)	21 (11.4)	17 (9.1)
125 001-150 000	26 (6.7)	13 (3.9)	10 (5.4)	11 (5.9)
150 001-200 000	40 (10.4)	12 (3.6)	8 (4.3)	5 (2.7)
≥200 000	33 (8.5)	6 (1.8)	5 (2.7)	0
Prefer not to answer or unknown	89 (23.1)	102 (30.6)	10 (5.4)	7 (3.8)
Maternal educational level				
Some high school	0	4 (1.2)	1 (0.5)	4 (2.2)
High school diploma or GED certificate	8 (2.1)	20 (6.0)	19 (10.3)	29 (15.6)
Some college	15 (3.9)	55 (16.5)	31 (16.8)	21 (11.3)
Vocational school certificate	1 (0.3)	12 (3.6)	6 (3.3)	2 (1.1)
Associate’s degree	4 (1.0)	14 (4.2)	14 (7.6)	13 (7.0)
Bachelor‘s degree	114 (29.5)	98 (29.4)	74 (40.2)	76 (40.9)
Master’s degree	135 (35.0)	55 (16.5)	31 (16.8)	29 (15.6)
Professional or doctoral degree	51 (13.2)	14 (4.2)	5 (2.7)	8 (4.3)
Prefer not to answer or unknown	58 (15.0)	61 (18.3)	3 (1.6)	4 (2.2)
ADOS-2^b^				
Social affect score				
Mean (SD)	2.3 (2.3)	13.6 (4.1)	3.1 (2.6)	13.8 (4.4)
Median (IQR)	2 (1-3)	14 (10-17)	3 (1-5)	14 (10-17)
Restricted and repetitive behavior score				
Mean (SD)	1.0 (0.9)	4.3 (1.8)	2.4 (1.6)	5.6 (1.4)
Median (IQR)	1 (0-2)	4 (3-6)	2 (1-4)	6 (5-7)
Total score				
Mean (SD)	3.3 (2.6)	17.9 (5.1)	5.5 (3.2)	19.4 (5.0)
Median (IQR)	3 (2-5)	18 (14-22)	5 (3-7)	20 (15-24)
Mullen Scales of Early Learning^c^				
Verbal ability age-equivalent score				
Mean (SD)	24.2 (5.6)	13.0 (6.2)	23.1 (8.0)	14.8 (7.7)
Median (IQR)	24 (20-28)	12 (8-16)	23 (16-28)	12 (10-18)
Nonverbal ability age-equivalent score				
Mean (SD)	24.8 (6.1)	19.0 (5.2)	27.3 (9.8)	20.7 (6.8)
Median (IQR)	24 (20-29)	19 (16-23)	25 (19-32)	20 (16-24)

Abbreviations: ADOS-2, Autism Diagnostic Observation Schedule, second edition; GED, general educational development.

^a^ Category percentages may sum to less than or greater than 100 due to rounding.

^b^ Includes 564 children (329 with autism diagnoses and 235 with nonautism diagnoses) in the discovery study and 255 children (186 with autism diagnoses and 69 with nonautism diagnoses) in the replication study. The standardized diagnostic protocol was sequential so that a child’s developmental history and scores on screenings dictated subsequent assessments. Further details provided in Reference Standard Diagnostic Assessment Procedures subsection of the eMethods in Supplement 1.

^c^ Includes 620 children (322 with autism diagnoses and 298 with nonautism diagnoses; 10 with missing nonverbal scores) in the discovery study and 251 children (183 with autism diagnoses and 68 with nonautism diagnoses) in the replication study. The verbal ability age-equivalent score, in months, was calculated as the mean of expressive and receptive language age-equivalent scores. The nonverbal ability age-equivalent score, in months, was calculated as the visual reception age-equivalent score. Further details provided in Reference Standard Diagnostic Assessment Procedures subsection of the eMethods in Supplement 1.

### Quality Control Indicators

Average calibration accuracy was within 1 degree of visual angle and did not differ significantly between diagnostic groups or study samples (eFigure 5 in Supplement 1). There were no significant between-group differences in duration of data collected (discovery: *t* = 1.48; *P* = .14; replication*: t* = 0.81*, P* = .42). Children with nonautism diagnoses did fixate (discovery: *t* = 4.97, *P* < .001; replication: *t* = 8.51, *P* < .001) and saccade (discovery: *t* = 6.75, *P* < .001; replication: *t* = 8.10, *P* < .001) significantly more, and blink less (discovery: *t* = 4.61, *P* < .001; replication: *t* = 4.08, *P* < .001), than children with autism, which was consistent with expected diagnostic differences in attention to and engagement with social cues in the environment^50^ that have been commonly noted in autism.^36^

### Primary End Points: Estimates of Diagnostic Accuracy

**Figure 2.**
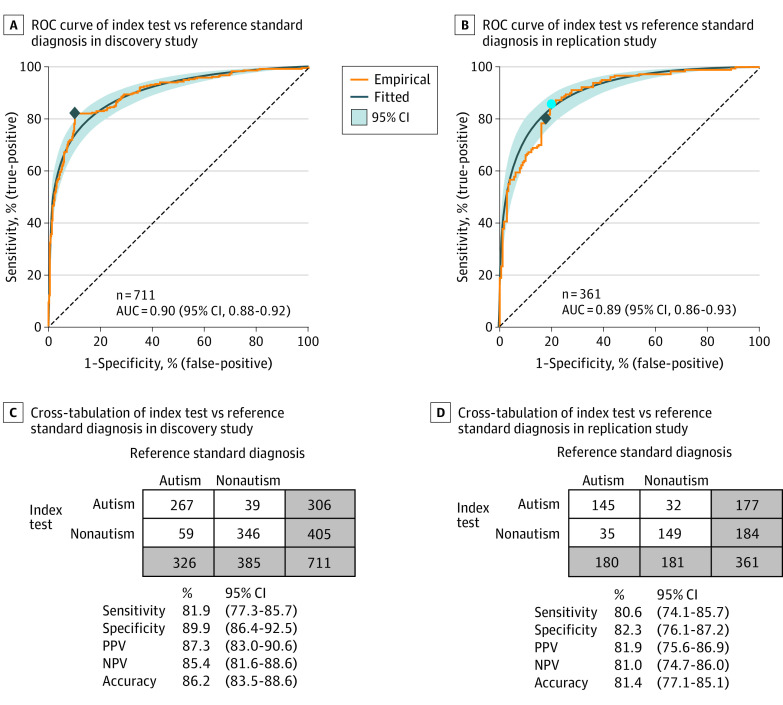
Diagnostic Performance Comparing Eye-Tracking–Based Measurement of Social Visual Engagement (Index Test) With Expert Clinical Diagnosis of Autism (Reference Standard) Performance among 711 children in the discovery study and 361 children in the replication study. A, The diamond represents the optimal test positivity threshold for the discovery study (Youden index). B, The test positivity threshold determined in the discovery study was fixed and applied independently in the replication study. The diamond represents the achieved sensitivity and specificity in the replication study using the test positivity threshold from the discovery study. The solid blue circle represents the post hoc theoretical optimal threshold. C, Tabulation corresponds to the diamond in panel A. D, Tabulation corresponds to the diamond in panel B. AUC indicates area under the curve; ROC, receiver operating characteristic. Negative predictive value (NPV) and positive predictive value (PPV) estimates reported here are calculated based on study sample prevalence.

Prespecified primary end point analyses measured the diagnostic accuracy of eye-tracking–based index test results in comparison with reference standard diagnosis. Results are shown in Figure 2 as receiver operating characteristic curves (panels A and B) and diagnostic cross-tabulations with performance measure estimates (panels C and D); underlying score distributions are plotted in eFigure 6 in Supplement 1. Index test performance had area under the curve statistics equal to 0.90 (95% CI, 0.88-0.92) in the discovery study and 0.89 (95% CI, 0.86-0.93) in the replication study. The test positivity threshold in the discovery study was selected to match the Youden index (represented by the diamond in Figure 2A). After discovery study determination, the test positivity threshold was fixed and applied in the replication study. Achieved sensitivity and specificity in the replication study are shown in Figure 2B (represented by the diamond, which corresponds to the cross-tabulation results and performance measure estimates in Figure 2D). Eye-tracking–based index test results predicted expert clinician reference standard diagnosis with sensitivity equal to 81.9% (95% CI, 77.3%-85.7%) and specificity equal to 89.9% (95% CI, 86.4%-92.5%) in the discovery study, and sensitivity equal to 80.6% (95% CI, 74.1%-85.7%) and specificity equal to 82.3% (95% CI, 76.1%-87.2%) in the replication study. Sensitivity, specificity, PPV, NPV, and accuracy did not differ significantly by sex (all with overlapping 95% CIs of performance estimates). Additional information regarding clinical outcomes is provided in the Clinical Outcomes of False Positives and Negatives subsection in eResults in Supplement 1.

### Secondary End Points: Measurements of Symptom Severity

**Figure 3.**
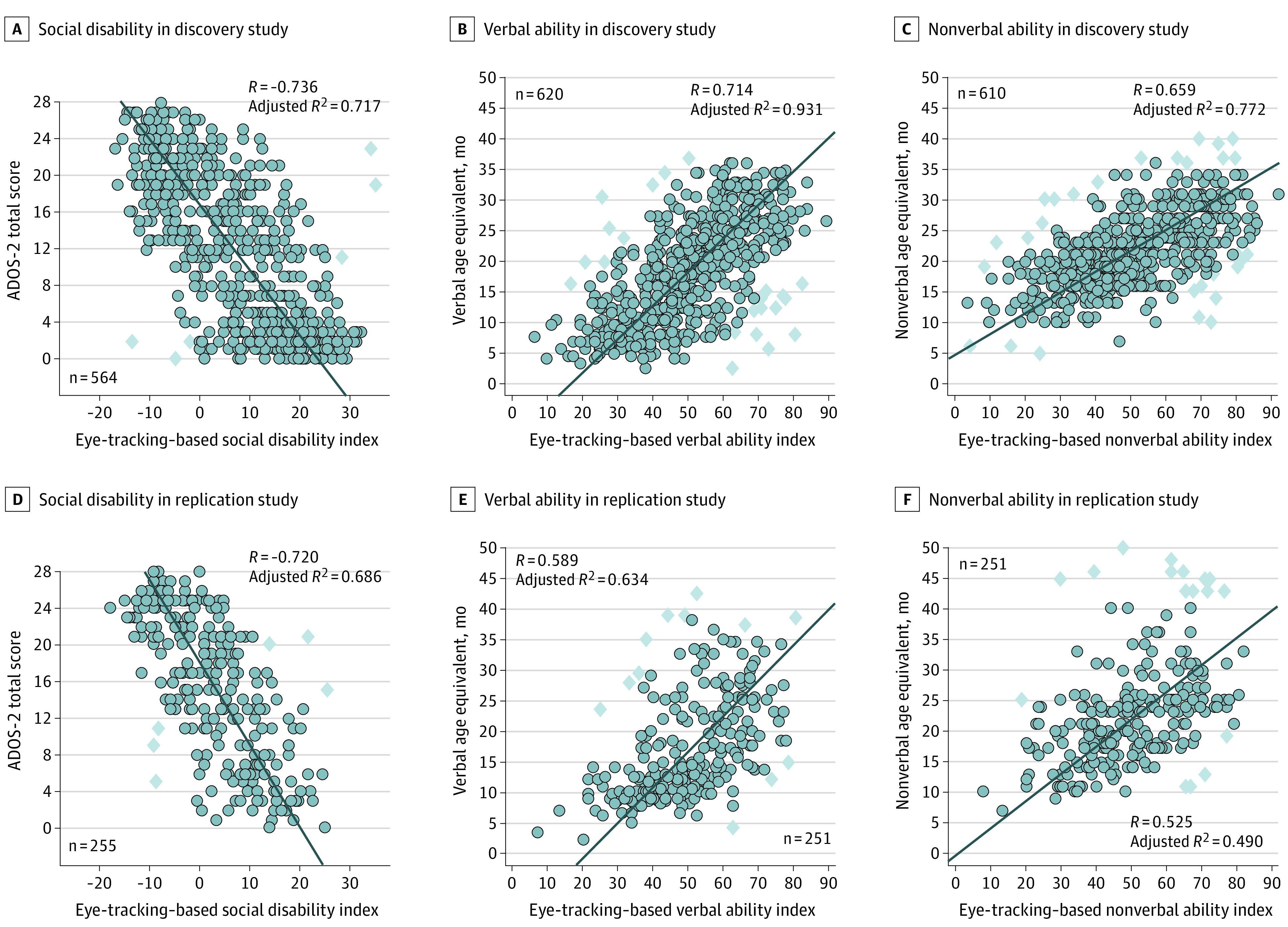
Convergent Validity Between Eye-Tracking–Based Measurement of Social Visual Engagement (Index Test) and Expert Clinician–Administered Standardized Assessments of Social Disability, Verbal Ability, and Nonverbal Cognitive Ability A, Discovery study correlation between eye-tracking–based indices of social disability versus children’s total scores on the Autism Diagnostic Observation Schedule, second edition (ADOS-2). B, Discovery study correlation between eye-tracking–based indices of verbal ability versus children’s verbal age equivalent scores as measured by the Mullen Scales of Early Learning (Mullen). C, Discovery study correlation between eye-tracking–based indices of nonverbal cognitive ability versus children’s nonverbal age equivalent scores as measured by the Mullen. D, E, F, Replication study correlations between eye-tracking–based indices and reference standard assessments. In all scatterplots, circles represent individual data and diamonds represent regression outliers (bivariate outliers identified using Cook distance and difference-in-fits regression diagnostic assessment). The adjusted *R*^2^ values were adjusted for measurement error variance of the reference standard (yielding percentage of reference standard nonerror variance explained by the index test). Additional information is provided in the Secondary End Point Analyses subsection of the eMethods in Supplement 1.

Prespecified secondary end point analyses measured the strength of association between eye-tracking–based indices and reference standard behavioral assessments of social disability, verbal ability, and nonverbal ability. Results are shown in Figure 3 as scatter plots with Deming regression fitted functions, Pearson *R* values, and adjusted *R*^2^ coefficients of determination (also summarized in eTable 1 in Supplement 1).

In the discovery study (Figure 3A-C), the correlation between the index test social disability index and the ADOS-2 total score was −0.74 (95% CI, −0.78 to −0.70), the correlation between the index test verbal ability index and the Mullen verbal age-equivalent score was 0.71 (95% CI, 0.67-0.75), and the correlation between the nonverbal ability index and the Mullen nonverbal age-equivalent score was 0.66 (95% CI, 0.61-0.70). In the replication study (Figure 3D-F), the correlation between the index test social disability index and the ADOS-2 total score was −0.72 (95% CI, −0.78 to −0.65), the correlation between the index test verbal ability index and the Mullen verbal age-equivalent score was 0.59 (95% CI, 0.50-0.77), and the correlation between the index test nonverbal ability index and the Mullen nonverbal age-equivalent score was 0.53 (95% CI, 0.43-0.62).

From the replication study, adjusted for reference standard measurement error, the eye-tracking–based social disability index accounted for 68.6% (adjusted *R*^2^ = 0.69; 95% CI, 0.58-0.79) of variance in ADOS-2 total scores. The verbal ability index accounted for 63.4% (adjusted *R*^2^ = 0.63; 95% CI, 0.48-0.79) of variance in Mullen verbal age-equivalent scores. The nonverbal ability index accounted for 49.0% (adjusted *R*^2^ = 0.49; 95% CI, 0.34-0.65) of variance in Mullen nonverbal age-equivalent scores.

In all comparisons, the strength of association between the eye-tracking–based indices and their respective expert clinician–administered assessments was high (*R* > 0.5), suggesting strong convergent validity between index and reference standard measures for social disability, verbal ability, and nonverbal ability. There were no significant differences in strength of association by sex. Participants with eye-tracking quality control indicator failures (8 of 719 in the discovery study and 9 of 370 in the replication study), with no results returned, are described further in eTables 2 and 3 in Supplement 1. Additional details and comparisons are available in the eResults in Supplement 1.

## Discussion

In 2 prospective double-blind diagnostic studies, the first a discovery study and the second a replication study, 1089 children were tested to measure the diagnostic performance of index test measurements of social visual engagement relative to reference standard expert clinical diagnosis of autism. Children aged 16 to 30 months (discovery study) and 16 to 45 months (replication study) were assessed by expert clinicians to test whether measurements of social visual engagement could accurately predict categorical diagnosis as well as dimensional levels of social disability, verbal ability, and nonverbal ability.

The results, reported in accordance with the STARD initiative,^27,28,29^ found that measurements of social visual engagement had 81.9% sensitivity and 89.9% specificity relative to expert clinical diagnosis of autism in the discovery study and 80.6% sensitivity and 82.3% specificity in the replication study. Sensitivity, specificity, PPV, NPV, and accuracy did not differ significantly between the discovery and replication studies, suggesting robust and replicable performance. In addition, measurements of social visual engagement were also predictive of children’s individual scores on gold standard behavioral assessments: measurements of social visual engagement effectively explained 68.6% of variance in individual levels of social disability (ADOS-2 total scores), 63.4% of variance in verbal ability (Mullen verbal age-equivalent scores), and 49.0% of variance in nonverbal cognitive ability (Mullen nonverbal age-equivalent scores).

These results suggest high convergent validity with reference standard assessments that otherwise require highly trained experts to spend multiple hours of assessment time per child. In contrast, for measurements of social visual engagement, biomarker data collection consisted of children watching videos (eFigure 1 in Supplement 1), with data collected on a standalone mobile eye-tracking device that was deployed in a clinic and operated by technicians with no required clinical or technical expertise (eFigure 2 in Supplement 1).

Once social visual engagement data are collected, although data processing and analysis are computationally intensive, they are also automated, deployed on cloud-based servers, and capable of returning a results report in less than 30 minutes. The index test is objective and quantitative and directly measures thousands of instances of children’s behavior for comparison with age-expected norms (examples are shown in eFigure 4 in Supplement 1). Data processing and analysis to derive diagnostic classification and indices of symptom severity are entirely automated, requiring no special expertise or eye-tracking knowledge on the part of clinicians.

It is important to note that the test results derived from measurements of social visual engagement are not intended to replace clinicians with expertise in developmental disabilities; to the contrary, a tool like this could be used by expert clinicians to aid in accurately and efficiently diagnosing autism as well as quantifying children’s strengths and vulnerabilities. Therefore, these results offer important opportunities to enhance health care system capacity and facilitate more rapid progress from the time of first concern to the start of individually appropriate services.^10^ While empirically supported services have their own access challenges, those challenges are not a reason to delay diagnosis or to delay initiation of supports for children and families.^51^

**Figure 4.**
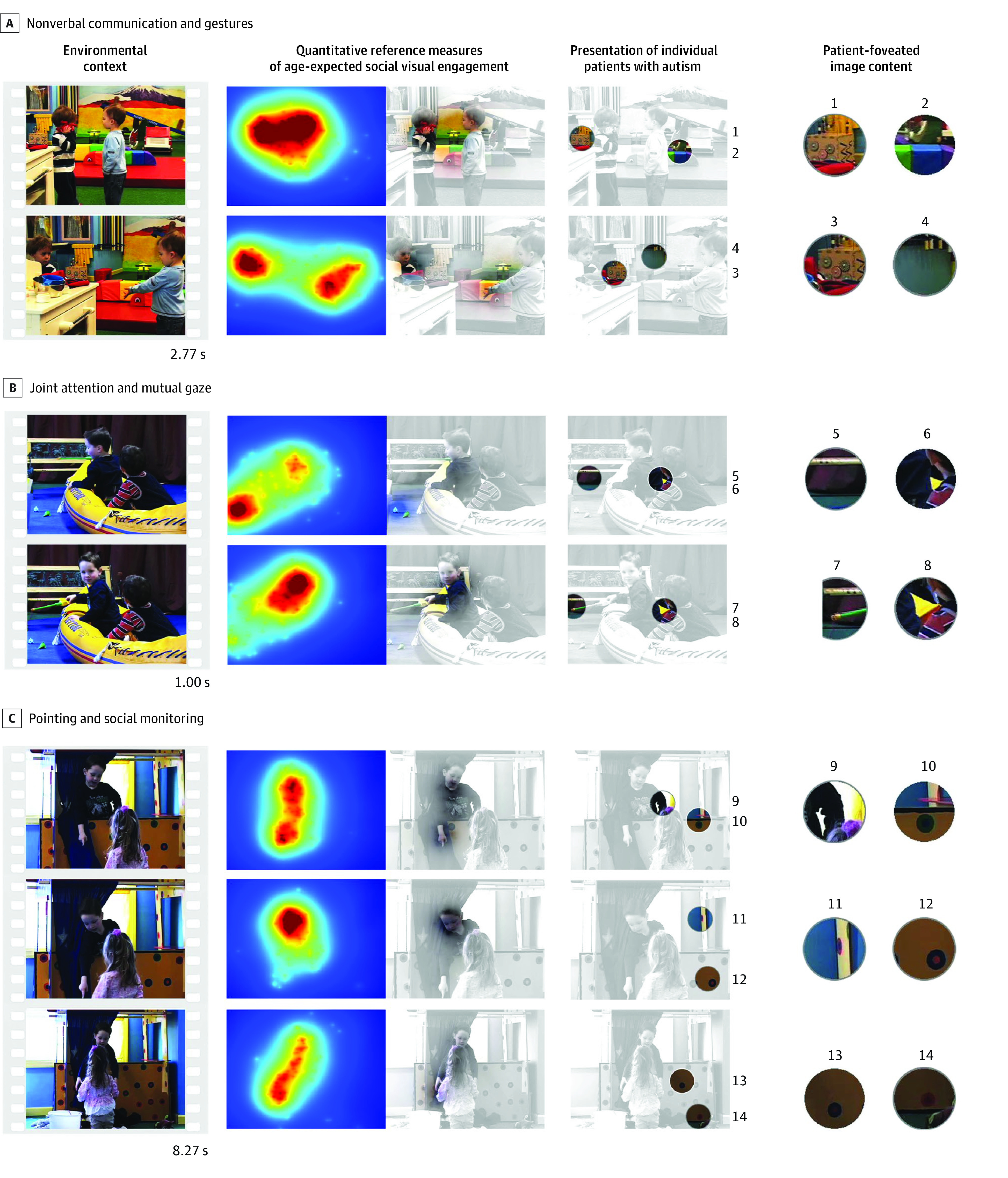
Performance-Based Measures of Children’s Individual Strengths, Vulnerabilities, and Opportunities for Skill Development Measurement of social visual engagement quantifies how a child engages with social and nonsocial cues occurring continuously within naturalistic environmental contexts (left column, shown as still frames from testing videos). In relation to those contexts, normative reference measures provide objective quantification of nonautism age-expected visual engagement (middle columns, shown as density distributions in both pseudocolor format and as color to grayscale fades overlaid on corresponding still frames). The age-expected reference measures can be used to measure and visualize patient comparisons, revealing individual strengths, vulnerabilities, and opportunities for skill building (right columns, sample patient data shown as overlaid circular apertures that encompass the portion of video foveated by each patient [each aperture spans the central 5.2 degrees of a patient’s visual field]). Children with autism present as engaging with toys of interest (1, 3, 5, and 7), color and contrast cues (2, 6, 8, and 9), and objects and background elements not directly relevant to social context (4 and 10-14). Elapsed times at the bottom right of still frames highlight the rapidly changing nature of social interaction in which many hundreds of verbal and nonverbal communicative cues are presented, each eliciting age-expected patterns of engagement and offering corresponding opportunities for objective quantitative comparisons of patient behavior.

Finally, the meaning of these measurements resides in what they quantify: repeated divergence from shared social experience with rapid accrual of atypical experience (Figure 4). Shared experience is the foundation for communication and social development. By quantifying the number, extent, and timing of divergence from shared experience, measurements of social visual engagement provide a transactional biomarker: direct objective measurements of a child’s unique biology transacting with specific environmental contexts. Those transactions are the building blocks of learning and brain development.^52^

Recognizing the transactional nature of this developmental process is a reminder that the emergence of disability is itself transactional, driven by genetic liabilities but also by atypical learning experiences that are correlated with those liabilities.^53^ Recognizing this provides 2 notable reasons for optimism. First, it reminds us that disability is a cocreation, a consequence of individual vulnerabilities transacting with particular environmental contexts, severely disabling in some contexts but less so or not at all in others.^54^ Fostering early intervention approaches and contexts that embrace difference and diversity while also augmenting individual adaptive skills is important to reducing disability and optimizing outcomes for all. Second, knowing that the unfolding of disability is transactional means that it can be measured as such to (1) identify children in need of support; (2) monitor specific behaviors and contexts that may exacerbate or ameliorate disability over time; and (3), ideally, intervene more successfully and treat specific individual manifestations and vulnerabilities for disability.

### Limitations

This study has several limitations. Clinical procedures were performed by a relatively small group of expert clinicians, and eye-tracking procedures were implemented under well-controlled laboratory conditions in the discovery study or with a single prototype standalone eye-tracking device in the replication study. The efforts in this study should be complemented by studies collecting reference standard and index test data at multiple different sites with multiple different clinical teams and eye-tracking devices.^55^ The results of this study should also be complemented by data quantifying repeatability and reproducibility variance in eye-tracking–based measurements.^56^ Previous studies^57,58^ have also noted expert clinical uncertainty in the reference standard diagnosis of autism in some children. Uncertainty in the reference standard sets an upper limit on the performance measures of any comparison test (eFigure 3 in Jones et al^55^). In the current study, we did not prospectively track expert clinician certainty of diagnosis in all children. Consequently, we were unable to analyze the effects of clinician certainty in the discovery or replication studies. This limitation was improved in a subsequent multisite study.^55^

## Conclusions

In 2 diagnostic studies of children aged 16 to 30 months with and without autism, objective measurements of social visual engagement were able to quantify diagnostic status and assess individual levels of social disability, verbal ability, and nonverbal ability. These findings suggest that objective measurements of social visual engagement can be used to aid in autism diagnosis and assessment.

## Notes

Supplement 1. **eFigure 1.** Example Video Stimuli and Coded Regions of Interest **eFigure 2.** Eye-Tracking Data Collection Device **eFigure 3.** Analysis of Dynamic Visual Scanning and Derivation of Attentional Funnels **eFigure 4.** Using Kernel Density Estimation to Derive Attentional Funnels and Quantify Dynamic Visual Scanning **eFigure 5.** Eye-Tracking Calibration Accuracy **eFigure 6.** Probability Density Functions of Individual Score Numeric Values Used to Make Index Test Categorical Determinations of Autism vs Nonautism, Plotted According to Reference Standard Expert Clinical Diagnosis in Discovery and Replication Studies **eTable 1.** Correlation of Eye-Tracking–Based Indices With Reference Standard Assessments **eTable 2.** Discovery Study Participants With Missing Eye-Tracking Data (Failed Quality Controls) **eTable 3.** Replication Study Participants With Missing Eye-Tracking Data (Failed Quality Controls) **eMethods.** Participants, Experimental Design, Experimental Procedures and Data Collection, Data Processing, and Data Analysis and Statistics **eResults.** Clinical Outcomes of False Positives and Negatives and Missing Data
eReferences

Supplement 2.
Data Sharing Statement
