# Supplementary material for: Development and Replication of Objective Measurements of Social Visual Engagement to Aid in Early Diagnosis and Assessment of Autism
Source: JAMA Netw Open. 2023 Sep 5;6(9):e2330145. doi: 10.1001/jamanetworkopen.2023.30145 (PMC10481232; doi:10.1001/jamanetworkopen.2023.30145)
Supplement: Supplement 1. — eFigure 1. Example Video Stimuli and Coded Regions of Interest eFigure 2. Eye-Tracking Data Collection Device eFigure 3. Analysis of Dynamic Visual Scanning and Derivation of Attentional Funnels eFigure 4. Using Kernel Density Estimation to Derive Attentional Funnels and Quantify Dynamic Visual Scanning eFigure 5. Eye-Tracking Calibration Accuracy eFigure 6. Probability Density Functions of Individual Score Numeric Values Used to Make Index Test Categorical Determinations of Autism vs Nonautism, Plotted According to Reference Standard Expert Clinical Diagnosis in Discovery and Replication Studies eTable 1. Correlation of Eye-Tracking–Based Indices With Reference Standard Assessments eTable 2. Discovery Study Participants With Missing Eye-Tracking Data (Failed Quality Controls) eTable 3. Replication Study Participants With Missing Eye-Tracking Data (Failed Quality Controls) eMethods. Participants, Experimental Design, Experimental Procedures and Data Collection, Data Processing, and Data Analysis and Statistics eResults. Clinical Outcomes of False Positives and Negatives and Missing Data eReferences [file jamanetwopen-e2330145-s001.pdf]

Supplementary Online Content

Jones W, Klaiman C, Richardson S, et al. Development and replication of objective measurements of social visual engagement to aid in early diagnosis and assessment of autism. *JAMA Netw Open*. 2023;6(9):e2330145. doi:10.1001/jamanetworkopen.2023.30145

eFigure 1. Example Video Stimuli and Coded Regions of Interest

eFigure 2. Eye-Tracking Data Collection Device

eFigure 3. Analysis of Dynamic Visual Scanning and Derivation of Attentional Funnels

eFigure 4. Using Kernel Density Estimation to Derive Attentional Funnels and Quantify Dynamic Visual Scanning

eFigure 5. Eye-Tracking Calibration Accuracy

eFigure 6. Probability Density Functions of Individual Score Numeric Values Used to Make Index Test Categorical Determinations of Autism vs Nonautism, Plotted According to Reference Standard Expert Clinical Diagnosis in Discovery and Replication Studies

eTable 1. Correlation of Eye-Tracking–Based Indices With Reference Standard Assessments

eTable 2. Discovery Study Participants With Missing Eye-Tracking Data (Failed Quality Controls)

eTable 3. Replication Study Participants With Missing Eye-Tracking Data (Failed Quality Controls)

eMethods. Participants, Experimental Design, Experimental Procedures and Data Collection, Data Processing, and Data Analysis and Statistics

eResults. Clinical Outcomes of False Positives and Negatives and Missing Data

eReferences

This supplemental material has been provided by the authors to give readers additional information about their work.

Table of Contents

eFigures and eTables

eFigure 1. Example Video Stimuli and Coded Regions of Interest……………………………. 3
eFigure 2. Eye-Tracking Data Collection Device..4
eFigure 3. Analysis of Dynamic Visual Scanning and Derivation of Attentional Funnels 5
eFigure 4. Using Kernel Density Estimation to Derive Attentional Funnels and Quantify Dynamic Visual Scanning 6
eFigure 5. Eye-Tracking Calibration Accuracy 7
eFigure 6. Probability Density Functions of Individual Score Numeric Values Used to Make Index Test Categorical Determinations of Autism vs Nonautism, Plotted According to Reference Standard Expert Clinical Diagnosis in Discovery and Replication Studies 8
eTable 1. Correlation of Eye-Tracking–Based Indices With Reference Standard Assessments...9
eTable 2. Discovery Study Participants With Missing Eye-Tracking Data (Failed Quality Controls)...10
eTable 3. Replication Study Participants With Missing Eye-Tracking Data (Failed Quality Controls)...11

eMethods..12
Participants 12
Experimental Design 15
Experimental Procedures and Data Collection 19
Data Processing..23
Data Analysis and Statistics 28

eResults...33
Clinical Outcomes of False Positives and Negatives 33
Missing Data 34

eReferences 36

eFigure 1. Example Video Stimuli and Coded Regions of Interest

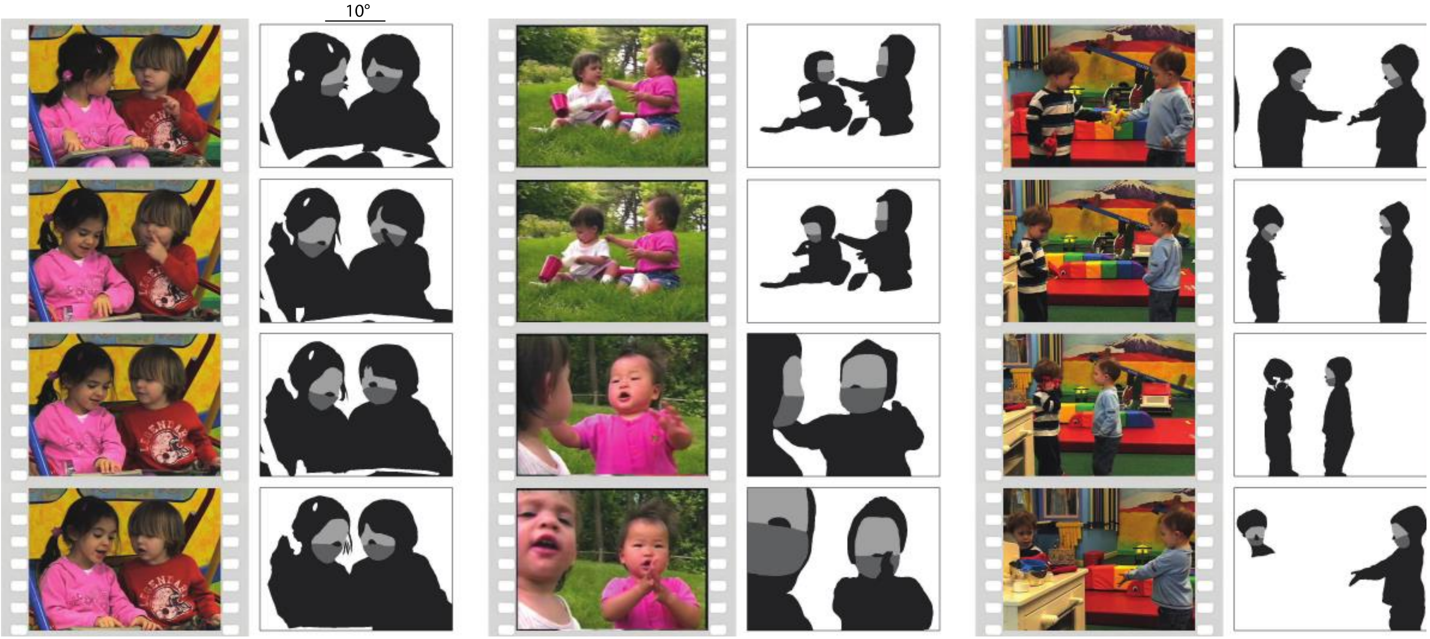

eFigure 1. Example video stimuli and coded regions-of-interest. Color panels show example still images from videos presented during eye-tracking data collection. Videos presented scenes of unscripted interaction between young children. **Grayscale panels** show example content regions (as coded for all frames of all video stimuli) corresponding to each still image in the adjacent color panel, shown as eyes, light gray; mouth, medium gray; body, dark gray; and object, white. Videos were audiovisual stimuli, 640x480 pixels in resolution, H.264 compression codec with 8-bit color, 44.1KHz audio, presented and analyzed at 30 frames per second. Scale bar marks degrees of visual angle when presented.

eFigure 2. Eye-Tracking Data Collection Device

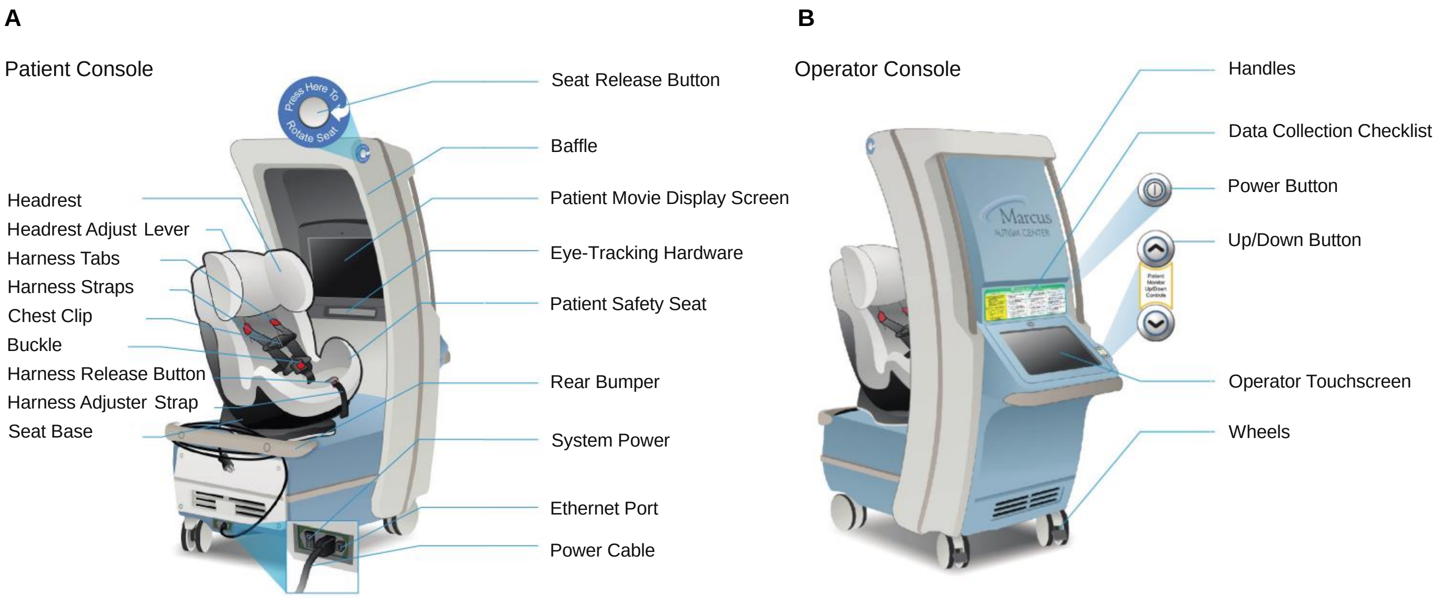

eFigure 2. Eye-tracking Data Collection Device. In the replication study, data were collected on a standalone eye-tracking data collection device located in a community clinic, as depicted above. (**A**) Patient console. Study participants sat in a child safety seat, watching videos on a movie screen as eye-tracking data were collected. (**B**) Operator console. The operator stood on the opposite side of the device, with a touchscreen display for overseeing the data collection process.

eFigure 3. Analysis of Dynamic Visual Scanning and Derivation of Attentional Funnels

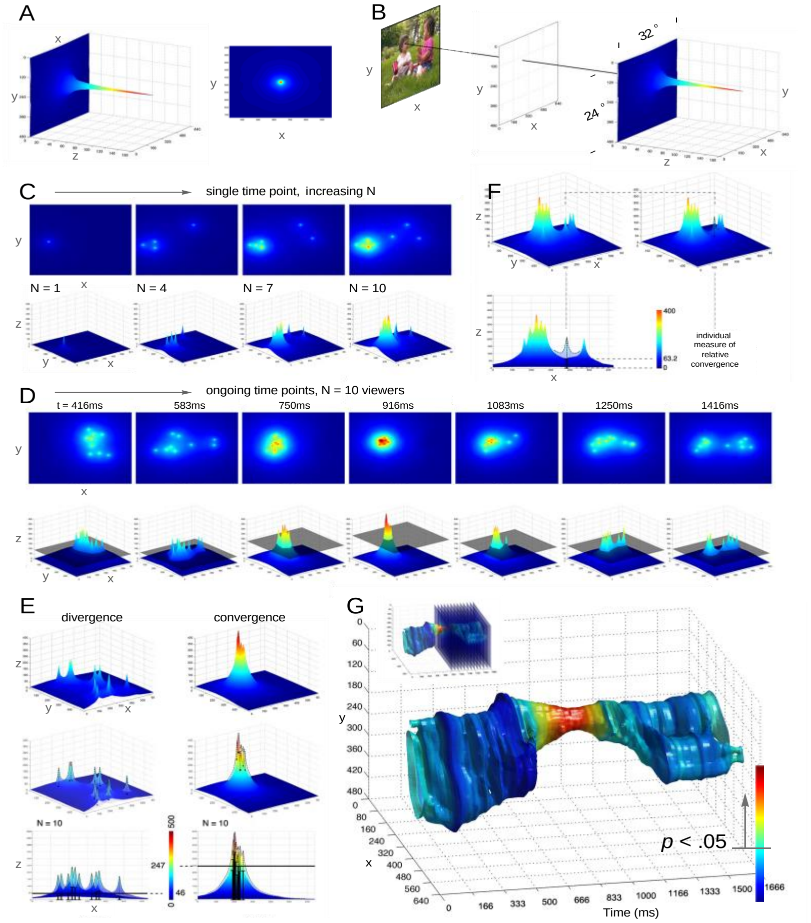

eFigure 3. Analysis of dynamic visual scanning and derivation of attentional funnels. We used kernel density estimation to quantify visual scanning for groups of viewers across the (X,Y) space of the presentation screen and throughout the duration of time series data. **(A - B)** We convolved the kernel function in (A) with coordinates of visual scanning (as in B). Peak of the kernel in (B) marks a single viewer's fixation location at one point in time. **(C)** We applied this process to time-locked data from all viewers in each group to derive kernel density estimates for the group as a whole (plots show top and oblique views for increasing number of viewers). **(D)** By repeating this process at each point in the time series, we quantified visual scanning as a dynamic probability density function. As shown in (D), distributions vary in time from widespread (panel at 416ms) to narrow (panel at 916ms). **(E)** We termed the extrema of these varying states "divergence" and "convergence" in visual scanning. To quantify these states statistically, we simulated kernel density estimates from randomized fixation data, yielding a measure of the probability that visual scanning would be widely divergent or narrowly convergent by chance alone. In relation to this statistical benchmark, we then measured the behavior of actual viewers. **(F)** Level of individual convergence was quantified by leave-one-out re-sampling (measuring each viewer in relation to the density estimate of the others). Median of all individual measures indexes the convergence or divergence of the group as a whole (bottom panels in part E). **(G)** An "attentional funnel" defined by statistically significant ($p < .05$) convergence in visual scanning. The funnels are instances of spatial and temporal entrainment of visual scanning, and were used to define experimental presses in which the visua l scanning of non-autism toddlers converged on targets that they perceived to be of high behavioral relevance, and against which the actions of comparison viewers could be mesured. X & Y axes are screen coordinates in pixels. Z axis is density estimate.

eFigure 4. Using Kernel Density Estimation to Derive Attentional Funnels and Quantify Dynamic Visual Scanning

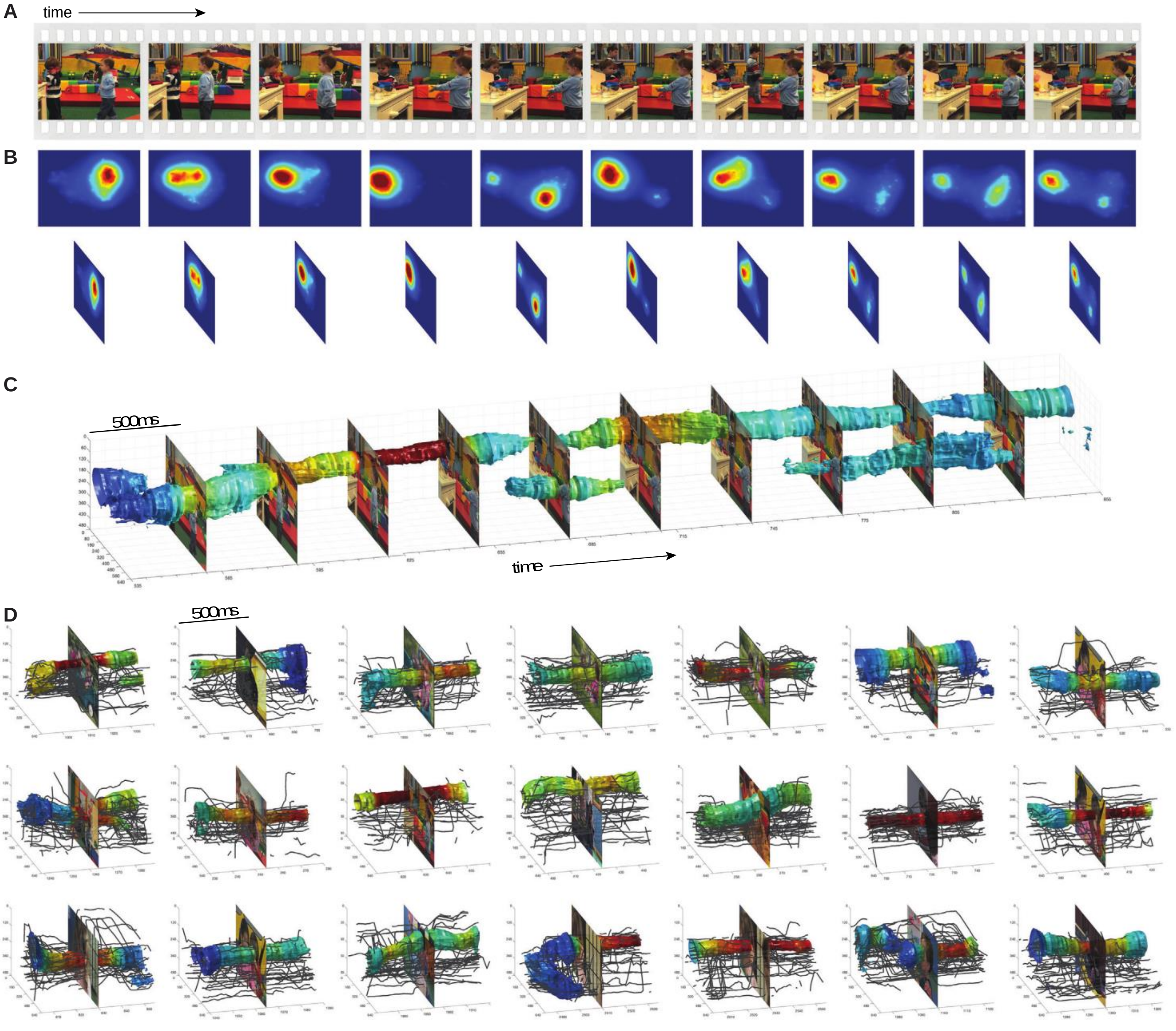

eFigure 4. Using kernel density estimation to derive attentional funnels and quantify dynamic visual scanning. (**A**) Example still frames from video testing stimuli. (**B**) Kernel density estimation quantifies visual scanning as a dynamic probability density function. Each plot in B is a measure of the probability of non-ASD children looking at the corresponding video still in A (shown in pseudocolor, with "hotter" color s denoting greater probability, and "cooler" colors denoting lesser probability). Rotating the data in B and plotting as a time-varying volume of data, (**C**) shows the sub-region with highest probability of being fixated over time (termed an "attentional funnel"). In the example plotted, the pr obability of non-ASD viewers fixating on specific onscreen content at specific moments in time becomes periodically very high, and the portion of te sting stimuli fixated—the target of social visual engagement at those moments—becomes very narrowly delimited: at times of greatest convergen ce in C, the portion of video content encompassed by the attentional funnel is less than 2.03% of the image's total area (e.g., 6222 of 307,200 pixels). The attentional funnel marks instances of non-ASD normative behavior against which the behaviour of other children can be compared and quantified. (**D**) shows representative plots in which the visual scanning of non-ASD children (shown by each funnel) converged significantly wh ile children with ASD fixated on alternate content (i.e., "diverged", shown as black lines marking the scanpaths of individual children with ASD). Each of these funnels is an example from one time point in the classification timeline. In each episode of between-group divergence in the discovery cohort data (episodes as exemplified in D), each viewer's fixation location is a measure for comparison with age-expected norms. These measures, at e ach moment in time, are the primary quantification of behavior used to compute each of the 4 eye-tracking based indices: diagnostic classification index, social disability index, verbal ability index, and nonverbal ability index.

eFigure 5. Eye-Tracking Calibration Accuracy

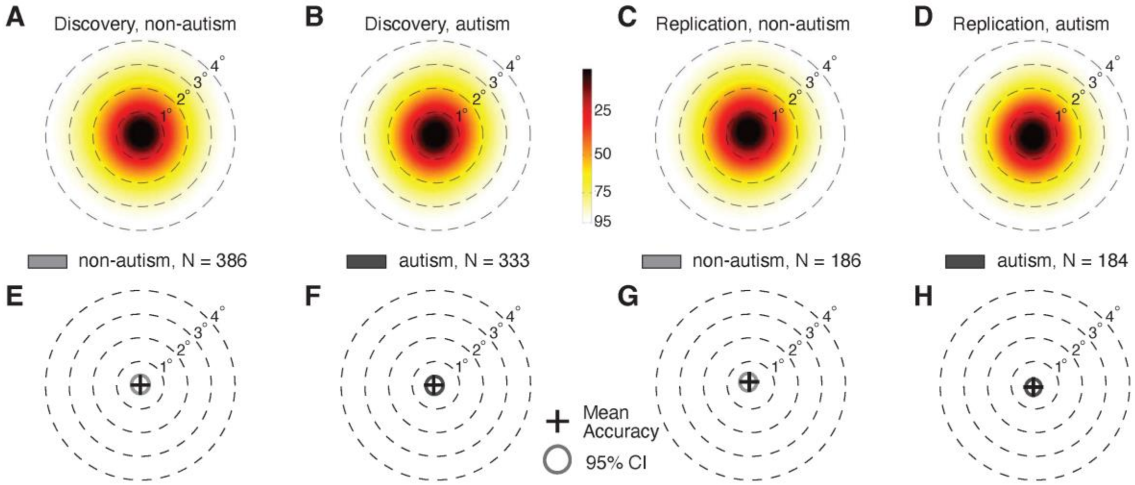

eFigure 5. Eye-tracking calibration accuracy. To test for between-study and between-group differences in quality of data, we compared calibration accuracy. (**A-D**) Total variance in calibration accuracy for the discovery study non-autism (A) and autism (B) groups, and for the replication study non-autism (C) and autism (D) groups. Plots show kernel density estimates of the distribution of measured fixation locations relative to calibration accuracy verification targets. (**E-H**) Average calibration accuracy for the discovery study non-autism (E) and autism (F) groups, and for the replication study non-autism (G) and autism (H) groups. A notable feature of these data is the similarity in autism versus non-autism fixation locations when a visual attention-getting target is presented (the calibration accuracy verification targets) in comparison with fixation locations in eFigure 4D, when the "target" of visual attention is not cued but is instead based on implicit social attribution and understanding of the social content. In eFigure 3D, the autism versus non-autism difference is marked, whereas here there is no statistical difference. In E-H, crosses mark the location of mean calibration accuracy, while annuli mark 95% confidence intervals (CI).

eFigure 6. Probability Density Functions of Individual Score Numeric Values Used to Make Index Test Categorical Determinations of Autism vs Nonautism, Plotted According to Reference Standard Expert Clinical Diagnosis in Discovery and Replication Studies

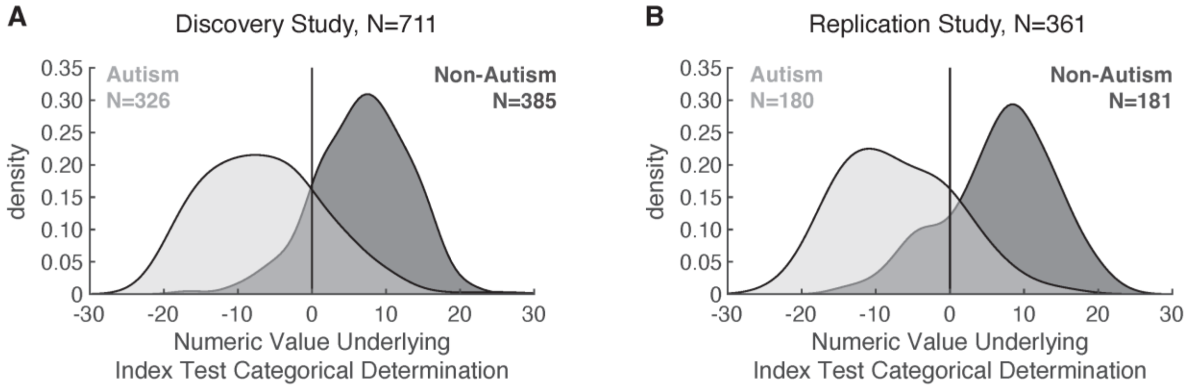

eFigure 6. Probability density functions of individual score numeric values used to make index test categorial determinations of autism versus non-autism, plotted according to reference standard expert clinical diagnosis in discovery and replication studies. Distributions of eye-tracking-based index test output values in (**A**) discovery study and (**B**) replication study. The prespecified test positivity threshold was determined in the discovery study, frozen, and applied in the replication study. Values less than or equal to 0 were considered to indicate autism. Distributions of scores for children with reference standard autism diagnosis are shown in light gray, distributions of scores for children with reference standard diagnosis of non-autism are shown in dark gray. Sample sizes reflect all children who completed eye-tracking data collection with data passing automated quality control indicators (711/719 in discovery and 361/370 in replication; the remaining participants, N=8 and N=9, respectively, failed quality control indicators; see Supplementary Tables 2 and 3 for further information).

eTable 1. Correlation of Eye-Tracking-Based Indices with Reference Standard Assessments

	Pearson *R*		Adjusted R^2	
	estimate	95% CI	estimate	95% CI
**Social Disability** [1]				
Discovery, no outliers, N = 557	-0.74	(-0.78, -0.70)	0.72	(0.65, 0.79)
Discovery, with outliers, N = 564	-0.69	(-0.73, -0.64)	0.64	(0.55, 0.73)
Replication, no outliers, N = 248	-0.72	(-0.78, -0.65)	0.69	(0.58, 0.79)
Replication, with outliers, N = 255	-0.66	(-0.73, -0.58)	0.58	(0.46, 0.70)
**Verbal Ability** [2]				
Discovery, no outliers, N = 598	0.71	(0.67, 0.75)	0.93	(0.84, 1.00)
Discovery, with outliers, N = 620	0.63	(0.58, 0.68)	0.73	(0.61, 0.85)
Replication, no outliers, N = 239	0.59	(0.50, 0.67)	0.63	(0.48, 0.79)
Replication, with outliers, N = 251	0.50	(0.40, 0.59)	0.46	(0.30, 0.63)
**Nonverbal Ability** [3]				
Discovery, no outliers, N = 588	0.66	(0.61, 0.70)	0.77	(0.68, 0.88)
Discovery, with outliers, N = 620	0.59	(0.54, 0.64)	0.61	(0.50, 0.73)
Replication, no outliers, N = 233	0.53	(0.43, 0.62)	0.49	(0.34, 0.65)
Replication, with outliers, N = 251	0.46	(0.36, 0.55)	0.38	(0.22, 0.55)

[1] - Correlation of *ADOS-2* Total Scores with eye-tracking-based index test Social Disability Indices. Sample sizes reflect total N for ADOS-2, with or without regression outliers; see Supplementary Materials: Reference Standard Diagnostic Assessment Procedures.

[2] - Correlation of *Mullen Scales of Early Learning* Verbal Age Equivalents (average of receptive and expressive language) with eye-tracking-based index test Verbal Ability Indices. Sample sizes reflect total N for Mullen scores, with or without outliers, see Supplementary Materials: Reference Standard Diagnostic Assessment Procedures.

[3] - Correlation of *Mullen Scales of Early Learning* Nonverbal Age Equivalents with eye-tracking-based index test Nonverbal Ability Indices. Sample sizes reflect total N for Mullen scores, with or without outliers, see Supplementary Materials: Reference Standard Diagnostic Assessment Procedures.

Adjusted R^2 values, with bootstrap 95% confidence intervals, are adjusted[66-68] for test-retest reliability of reference standard assessments[26,38]. See Supplementary Materials: Secondary Endpoint Analyses. CI = confidence interval.

eTable 2. Discovery Study Participants with Missing Eye-Tracking Data (Failed Quality Controls)

ID	Session Comments	QCI_calibrationAccuracy	QCI_durationOfDataCollected	QCI_percentageTimeSpentWatching	QCI_numberOfVideosWatched	QCI_numberOfVideosExcluded	QCI_signalToNoiseRatio [1]	QCI_saccadePercentageRate [1]
01806	none	PASS	FAIL	FAIL	FAIL	FAIL	N/A	N/A
07918	Child got really squirmy through the last few clips.	PASS	FAIL	FAIL	FAIL	FAIL	N/A	N/A
04383	Child tilted head way to the side for some of the clips.	PASS	FAIL	FAIL	FAIL	FAIL	N/A	N/A
04910	Wasn't interested in the check calibs.	PASS	FAIL	FAIL	FAIL	FAIL	N/A	N/A
02805	Child tried to eat his shoe during the session.	PASS	FAIL	FAIL	FAIL	FAIL	N/A	N/A
06077	very mobile during testing.	PASS	FAIL	FAIL	FAIL	FAIL	N/A	N/A
10666	Child Movement: Sometimes. Did not get through the whole playlist.	PASS	FAIL	FAIL	FAIL	FAIL	N/A	N/A
10797	Child Movement: Sometimes. Child watched about half of the playlist, then got fussy.	PASS	FAIL	FAIL	FAIL	FAIL	N/A	N/A

[1] - The eye movement signal-to-noise ratio quality control indicator (QCI) and the saccade percentage and rate QCI (final 2 columns) were further developed in the discovery study for use in replication, and so were not calculated in the original analysis of the discovery study. N/A = not applicable.

eTable 3. Replication Study Participants with Missing Eye-Tracking Data (Failed Quality Controls)

ID	Session Comments	QCI_calibrationAccuracy	QCI_durationOfDataCollected	QCI_percentageTimeSpentWatching	QCI_numberOfVideosWatched	QCI_numberOfVideosExcluded	QCI_signalToNoiseRatio	QCI_saccadePercentageRate
10448	Child Movement: Always. ChildEatingOrDrinking: yes. Repeated "done" until mom decided to end session.	FAIL	PASS	FAIL	PASS	PASS	PASS	FAIL
11721	Child Movement: Always. Child did not settle in chair.	PASS	FAIL	FAIL	FAIL	FAIL	PASS	FAIL
10458	Child Movement: Always. Child never settled in >10minutes. We did get calibration done.	PASS	PASS	FAIL	PASS	PASS	FAIL	FAIL
00087	Child Movement: Often. childEatingOrDrinking: yes.	PASS	PASS	FAIL	PASS	FAIL	PASS	FAIL
00189	Child Movement: Often. kicking and readjusting.	PASS	PASS	FAIL	PASS	FAIL	PASS	FAIL
00387	Child Movement: Never. childEatingOrDrinking: no.	PASS	PASS	FAIL	PASS	PASS	PASS	FAIL
10956	childEatingOrDrinking: yes.	PASS	PASS	FAIL	PASS	PASS	PASS	FAIL
11596	Child Movement: Often	FAIL	PASS	FAIL	PASS	FAIL	PASS	FAIL
14404	Child Movement: Always. childEatingOrDrinking: no. Way too much movement. Ended early.	PASS	PASS	FAIL	PASS	PASS	PASS	FAIL

eMethods. Participants, Experimental Design, Experimental Procedures and Data Collection, Data Processing, and Data Analysis and Statistics

This research was conducted in the Marcus Autism Center, Children's Healthcare of Atlanta, Department of Pediatrics, Emory University School of Medicine, and in the Intellectual and Developmental Disabilities Research Center at Washington University in St. Louis. The research protocol was approved as non-significant risk by the Emory University Institutional Review Board, and by the Washington University School of Medicine Human Research Protection Office. The data collected were used for research purposes only with no relationship to clinical care. Parents and/or legal guardians of all participants gave informed consent prior to participation. Families were free to withdraw at any time. Research was supported by grants from the Marcus Foundation; the Whitehead Foundation; the Georgia Research Alliance; the National Institute of Mental Health, MH100029 (AK, WJ); and by the National Institute of Child Health & Human Development, HD068479 (JNC).

As described in greater detail in the following sections, children were shown video scenes of naturalistic peer interaction; visual scanning was measured with eye-tracking equipment (ISCAN, Inc. and Sensomotoric Instruments, GmbH); analysis of eye movements and coding of fixation data were performed with software written in MATLAB; and data acquisition and processing were performed by experimenters blind to clinical assessment data. Details of participants, experimental design, data collection procedures, data processing, and data analysis and statistics are provided below.

Participants

Participating children were consecutive study referrals comprising a broad range of initial developmental concerns, from children having no developmental concerns to children for whom a parent or healthcare provider had concerns of autism spectrum disorder (ASD) or other general developmental delays. Participants were not intended to be representative of a general population sample with expected ASD prevalence of ~1/36, nor was the study designed to test utility of this tool as a screener. To develop measurements of social visual engagement that could aid in the diagnosis of ASD and aid in assessment of individual abilities and disabilities, children with a broad range of initial developmental concerns, enriched for concerns about ASD, were recruited.

Recruitment

Recruitment occurred via flyers and advertisements; via collaborations with local parent groups; via information in specialty diagnostic centers; and via information in large pediatric practices. In the replication study, a portion of the sample was epidemiologically ascertained through the Missouri Family Register (MFR), a birth records registry maintained by the WUSM Department of Psychiatry in collaboration with the State of Missouri as described in detail in Marrus, N. *et al.* Rapid video-referenced ratings of reciprocal social behavior in toddlers: a twin study. *J. Child Psychol. Psychiatry* **56,** 1338–1346 (2015). For any twins from the University of Washington in St. Louis site, only data from one randomly selected twin (i.e., excluding all data from the other sibling) was included in this study in order to ensure independent observations.

Inclusion and Exclusion Criteria

Participants were eligible for study participation if they met the following inclusion criteria:

- no clinically significant hearing or visual impairments (e.g., congenital deafness, blindness, nystagmus);
- no previously-diagnosed genetic conditions associated with ASD-related symptomatology (e.g., not known to have Fragile X, Rett Syndrome);
- generally healthy at time of participation with no acute illness (by parent report);
- chronological age between
    - 16 and 30 months (discovery study), or
    - 16 and 45 months (replication study);
- gestational age at birth
    - at or after 37 weeks gestational age (discovery study), or
    - at or after 32 weeks gestational age (replication study).

Participants were ineligible for participation if they met any of the following exclusion criteria:

- significant hearing loss or visual impairment (e.g., congenital deafness, blindness, nystagmus);

- previously-diagnosed genetic conditions associated with ASD-related symptomatology (e.g., known to have Fragile X syndrome, Tuberous Sclerosis, Rett Syndrome, or genetic mutations with high likelihood of ASD-related symptomatology);
- previously-diagnosed neurological conditions affecting visual or auditory acuity that could not be or had not been corrected (e.g., congenital nystagmus);
- history of head trauma with loss of consciousness;
- non-febrile seizure disorder;
- acute illness (e.g., fever, vomiting).

Discovery Study

The discovery study comprised N=719 children between 16-30 months of age. Mean age of the entire discovery cohort was 22.4 months with a standard deviation (SD) of 3.6 months. The purpose of the discovery study was to provide a data set that could be used to develop robust algorithms for diagnostic classification and prediction of symptom severity. To that end, discovery study participation was restricted to children who successfully completed the lab-based eye-tracking data collection session and could provide high quality eye-tracking data to algorithm development. (No such restrictions were placed on participation in the independent replication study sample.) In the discovery study, sixty-four additional children attempted but did not successfully complete the lab-based data collection session; no data from these children were used. The 719 children who did successfully complete the lab-based eye-tracking data collection session formed the discovery study cohort.

Based on reference standard diagnosis, the discovery study comprised N=386 non-ASD and N=333 ASD (main text **Table 1** provides participant characterization and demographic data according to the reference standard outcome groups). Please see “Experimental Procedures and Data Collection: Reference Standard Diagnostic Assessment Procedures” section for details about reference standard diagnostic assessments and criteria for diagnostic group membership.

Replication Study

The replication study cohort was an independent sample (recruited, enrolled, and participating independently of the discovery study, i.e. not a “left-out” or withheld subsample) and comprised N=370 children between 16-43 months of age. Mean age of the entire replication cohort was 25.4 months with a standard deviation (SD) of 6.0 months. In the replication study, criteria for age and gestational age at birth were broadened slightly, by design, in order to test effectiveness with less restrictive criteria. Eligible referrals were enrolled consecutively. There were no restrictions or criteria for successful completion of data collection (as had been required in the discovery study); eligible children were offered enrollment, and those enrolled were tested, with any missing or failed data collection sessions recorded as such. Of 380 eligible children, 10 did not enroll (8 declined to enroll while 2 did not present for testing at the scheduled study appointment and could not be reached for follow-up), leaving 370 replication study participants, of whom 9 failed in data collection (~2.4% failure rate), yielding 361 children for endpoint analyses (main text **Figure 1**).

Based on reference standard diagnosis, the replication study comprised N=184 non-ASD and N=186 ASD (main text **Table 1** provides participant characterization and demographic data according to the reference standard outcome groups). Please see “Experimental Procedures and Data Collection: Reference Standard Diagnostic Assessment Procedures” section for details about reference standard diagnostic assessments and criteria for diagnostic group membership.

Experimental Design

Study Objectives

The overarching study objectives were (1) to measure the diagnostic accuracy of an eye-tracking-based index test for ASD in comparison with clinical reference standard diagnosis, as well as (2) to measure the strength of association between eye-tracking-based indices of social disability, verbal ability, and nonverbal cognitive ability in comparison with clinical reference standard assessments of the same. More specifically, this study was conducted as an efficacy study to test the efficacy of eye-tracking-based assays to diagnose and measure levels of ASD symptom severity in 16-30-month-old children. For terminological consistency[1], the target condition (condition of interest) was ASD. The intended use

population was children 16-30 months old. The expected frequency of use of the eye-tracking-based index test was 1 successful session. The physiological use of the testing device was non-invasive measurement of child behavior (as measured by visual scanning) while watching age-appropriate, child-friendly video scenes. The measurement assay methodology was near-infrared video-based eye-tracking, and the intended use (purpose of measurement) was to assess presence and severity of ASD.

The discovery study was intended to develop and test algorithms for classification and measurement of symptom severity, while the replication study was intended as an independent test of performance. In both discovery and replication studies, the prespecified primary study endpoints were measured as sensitivity and specificity of the eye-tracking-based index test relative to the reference standard (clinician best estimate diagnosis using standardized instruments), while the prespecified secondary study endpoints were measured as correlation between eye-tracking-based indices and their clinical gold standard behavioral assessment counterparts (eye-tracking-based social disability index to ADOS total score; eye-tracking-based verbal ability index to Mullen verbal age equivalent score; eye-tracking-based nonverbal ability index to Mullen nonverbal age equivalent score).

Study Design

Both studies were designed as prospective, double-blind, within-subject comparisons of eye-tracking-based measures relative to reference standard diagnosis and developmental assessments. The overall design had two parts embodied in each of the two planned studies: discovery (for developing the testing algorithms and establishing diagnostic thresholds and quality control criteria) and replication (for independently testing the performance of algorithms and thresholds established in discovery). Consecutive participants meeting inclusion and exclusion criteria and whose parents or legal guardians provided informed consent were enrolled in the study. The clinical assessment procedures and eye-tracking data collection were completed concurrently during 1-2 days of participant testing (duration of participant testing was determined by parents and clinicians on the basis of reference standard clinical assessment procedures and clinical best practice regarding whether a child was able to complete all reference standard assessment procedures in either 1 or 2 days). Reference standard assessments and index test eye-tracking happened concurrently (i.e., a reference standard assessment followed by the

single session of eye-tracking index test data collection or vice versa), with no time interval nor clinical interventions between the index test and reference standard. The study was designed as an observational study with no impact to best practice clinical standard of care for participants. To that end, neither a child's parents nor the expert clinical staff were informed of a child's eye-tracking results, so that only clinical best practice standardized assessments and reference standard diagnosis were used clinically or communicated to parents.

Design Decisions to Minimize Bias

Lijmer et al[2] describes sources of potential bias in studies of diagnostic accuracy that should be controlled in order to eliminate or minimize effects of that bias. To that end, both discovery and replication studies were prospective, double-blind comparisons of the index test and reference standard in a consecutive series of participants from the intended use population. For the discovery study sample, given the goal of developing robust algorithms for diagnostic classification and prediction of symptom severity, participation was restricted to children who successfully completed a lab-based eye-tracking data collection session and could provide high quality eye-tracking data to algorithm development (there were no such restrictions in the replication study). This was a necessary design choice made to develop algorithms on the basis of high quality data, that could then be tested against more variable, real-world data (in the independent replication sample).

To minimize data collection biases, all data were collected prospectively. To minimize spectrum bias, both studies enrolled a wide spectrum of participants comprising a broad range of initial developmental concerns, from children having no developmental concerns, to children for whom a parent or healthcare provider had concerns, to children who were epidemiologically ascertained from the general population irrespective of concerns. In order to minimize selection bias, all participants in the replication study were enrolled as consecutive study referrals (though as noted above, discovery study participation was restricted to participants who successfully completed lab-based eye-tracking data collection session and could provide high quality eye-tracking data). Neither the discovery study nor the replication study pre-stratified enrollment groups by risk (i.e., creating a case-control study); participants were enrolled as consecutive study referrals and tested blind relative to reference standard diagnostic outcome or clinical

assessment measures. To minimize verification bias, participants underwent the same study procedures (both index test and clinician best estimate diagnosis). As described below in Reference Standard Diagnostic Assessment Procedures, there was variability as to which standardized assessments were required along with the reference standard diagnosis: if a child's scores on screeners for autistic symptomatology were low and the expert clinician felt there were no concerns for ASD, then the ADOS-2 was not administered; if there were any concerns about potential social disability, then the ADOS-2 was administered.

In order to eliminate interpretation bias, results of both the index test and reference standard were gathered, analyzed, and interpreted without knowledge of the comparison test, and processing and analysis of the eye-tracking data were fully automated. The study design was double-blind, with all staff involved with data gathering, analysis, and interpretation of index test results blind to reference standard results and all staff involved with data gathering, analysis, and interpretation of reference standard results blind to index test results. Neither parents nor expert clinical staff were informed of a child's index test eye-tracking results; only the reference standard diagnosis and assessment measures were used clinically.

Testing Stimuli

Children were shown video scenes of social interaction (See **eFigure 1** for examples). These "peer-play" videos presented scenes of unscripted peer interaction and have been used in other published studies of social visual engagement[3,4]. Scenes were selected from video of children at play, filmed with the written informed consent of the children's parents. Scenes presented peer interactions showing (for example) children sharing a snack; toddlers playing together in a shallow pool; two infants babbling; and children learning to share.

Fourteen video scenes were presented, each approximately 54.0 seconds in mean duration (SD=21.5 seconds), ranging from 21.7 seconds to 1 minute 29.7 seconds (summing to approximately 12 minutes 35.5 seconds in total video duration). Videos were presented as full-screen audiovisual stimuli in 32-bit color; at 640x480 pixels in resolution; at 30 frames per second; with mono channel audio sampled

at 44.1 kHz. Stimuli were sound and luminosity equalized, and piloted prior to the start of study in order to optimize engagement for toddler viewers.

Experimental Procedures and Data Collection

Index Test Eye-Tracking Data Collection Procedures

Data collection procedures followed those reported in [3,4]. Children were accompanied at all times by a parent or primary caregiver. To begin the eye-tracking data collection session, the child and caregiver entered the testing room while a children's video played on the display monitor (the videos played at the start of the session were not testing stimuli but were instead common children's videos used to attract the child's attention during initial setup). The child was buckled into a child safety seat. During testing, both experimenter and parent were out of view from the child but were able to monitor the child by means of a second video camera (streaming a wide angle image of the child's face and body).

Two types of eye-tracking data collection equipment were used to measure visual scanning and fixation data: lab-based testing equipment with eye-tracking hardware and software created by ISCAN Inc. (Woburn, Massachusetts), and a standalone eye-tracking data collection unit designed in-house (**eFigure 2**), using eye-tracking hardware and software created by SensoMotoric Instruments, GmbH (Teltow, Germany). The standalone eye-tracking data collection unit was used in the replication study. In both cases, the eye-tracking technology was video-based and used a dark pupil/corneal reflection technique for collecting data. In the lab setting, the eye-tracking camera was remotely mounted, concealed from the child's view behind an infrared filter in the wall panel. In the standalone eye-tracking data collection unit, the eye-tracking camera was also remotely mounted behind an infrared filter in a custom casement covering the movie display screen and eye-tracking hardware. In the lab setting, viewers' eyes were approximately 30 inches (76.2 centimeters) from a 20" diagonal display monitor, which subtended an approximately 24.0° x 32.0° portion of each viewer's visual field. In the standalone unit, viewers' eyes were approximately 23 inches (58.8 centimeters) from an 18.5" diagonal display monitor, with video content subtending an approximately 22.1° x 29.4° portion of each viewer's visual field. Eye position relative to display monitor was standardized for each child by vertically adjusting the

seat height (laboratory testing setting) or the display monitor (standalone eye-tracking unit); horizontal distance was fixed in both cases.

To begin the process of data collection, after the child was comfortably watching the children's video, calibration targets were presented onscreen by the experimenter. This was done via automated software that paused the playing video and presented a calibration target on an otherwise blank background. A five-point calibration procedure was used in the lab setting and a two-point calibration procedure in the standalone data collection unit, presenting spinning and/or flashing points of light as well as cartoon animations, ranging in size from 1.0° to 1.5° of visual angle, all with accompanying sounds. The calibration routine was followed by verification of calibration in which more animations were presented at five on-screen locations. Throughout the remainder of the testing session, animated targets (as used in the calibration process) were shown between experimental videos to measure possible drift in accuracy. In this way, accuracy of the eye-tracking data was verified before beginning experimental trials and was then repeatedly checked between video segments as the testing continued. For successful calibration, all participants were required to be capable of saccading to a visual target and maintaining stable foveation (less than 5°/second of drift when fixating).

Any failures of data collection were noted in the data collection software (subsequently stored in the research database along with all collected data). Reasons for failed data collection were equipment failure (computer hardware failure; operator error during set-up or operation) and child non-compliance (child fell asleep during testing; child cried during testing; child moved too much during testing; or child had a tantrum due to general fatigue or dislike of child safety seats). Sessions were stopped before a child completed watching all stimuli if the child fell asleep or became too fussy to watch the videos. Please see **eTables 2** and **3** and Supplementary Results: Missing Data section below for additional information about missing data.

Reference Standard Diagnostic Assessment Procedures

Reference standard clinician best estimate diagnosis[5] of ASD or non-ASD was made on the basis of the totality of information gained from direct behavioral assessments as well as developmental questionnaires and parent interviews. The primary expert clinician conducting the evaluation made the

final diagnostic determination. In case the primary expert ASD clinician was uncertain of a child's diagnostic classification, a second expert clinician was on site, with the opportunity to review all case information and discuss the case with the primary clinician to help disambiguate final determination and reach best estimate diagnostic consensus.

Direct behavioral assessments included the *Autism Diagnostic Observation Schedule, 2nd Edition*[6] (ADOS-2, a standardized, validated instrument that measures the presence of autistic social disability through assessment of a child's communication, social interaction, play, and restricted and repetitive behaviors) and the *Mullen Scales of Early Learning*[7] (Mullen, a standardized assessment of a child's nonverbal, verbal, and motor abilities). The ADOS-2 was administered by an expert ASD clinician with research reliability ascertained by a certified ADOS-2 trainer. The ADOS-2 Overall Total score was used to quantify severity of autistic social disability in the pre-specified secondary analyses. The Mullen was also administered by the primary expert ASD clinician. Mullen Age Equivalent scores, in months, in the visual reception (nonverbal) domain as well as in receptive and expressive language (verbal) domains, were used to quantify severity of ASD in the pre-specified secondary analyses (eye-tracking-based verbal ability index was compared to the average of receptive and expressive language age equivalent).

Parent questionnaires and developmental inventories were administered covering prenatal, perinatal, general health, and developmental history. At Washington University, the *MacArthur-Bates Communicative Development Inventories (CDI*) was administered as a broad screener for language delays, and the *Early Screening of Autistic Traits Questionnaire (ESAT*) was administered as a screener for autistic symptomatology.

In discovery and replication studies, if a child's developmental history had no concerns and scores on screeners for autistic symptomatology were low and the expert clinician felt there were no concerns for ASD, then the ADOS-2 was not administered; if there were any concerns about potential social disability, then the ADOS-2 was administered. Consequently, in the discovery study, all 333 participants with ASD were administered an ADOS-2 (329/333 have corresponding ADOS-2 data in the data set, as noted in main text **Table 1**, with 4 missing values due to data entry errors), while only 235/386 non-ASD participants have ADOS-2 data (N=564 total ADOS-2 assessments in the discovery

study). Likewise, in the replication study, all 186 participants with ASD were administered an ADOS-2 and have corresponding values, but only 69/184 non-ASD participants have ADOS-2 data (N=255 total ADOS-2 assessments in the replication study).

Following current best clinical practice, a parent conference to inform parents of the child's reference standard diagnosis was conducted for all participants, coinciding with the participant's study completion. No results from the eye-tracking index test procedure were made available to parents, clinicians, technicians, or clinical study staff. The expert ASD clinician who assessed the child and determined the child's ASD status conducted the conference.

All children diagnosed as having ASD received an expert clinician best estimate diagnosis of autism spectrum disorder based on the totality of information available about the child and in accordance with DSM-5 criteria for ASD. Non-ASD participants did not receive an expert clinician best estimate diagnosis of ASD, but did have a broad range of developmental outcomes at time of testing, ranging from no developmental delays (as measured via the *Mullen Scales of Early Learning* and operationalized as having neither one single score lower than 2 SD's from the normative average, nor 2 scores lower than 1.5 SD's from the normative average) to children who—without meeting criteria for ASD—were diagnosed with other developmental delays (measured either via the *Mullen*, operationalized as having one single score lower than 2 SD's from the normative average or 2 scores lower than 1.5 SD's from the normative average, or via scores equal to or lower than the 5th percentile on the *MacArthur-Bates Communicative Development Inventories*). In the discovery study, 322 participants with ASD have Mullen data, but only 298/386 non-ASD participants have Mullen data (N=620 total Mullen verbal assessments in the discovery study, N=610 nonverbal). Likewise, in the replication study, 183 participants with ASD have Mullen data but only 68/184 non-ASD participants have Mullen data (N=251 total Mullen assessments in replication).

Clinician best-estimate diagnosis[5] was chosen as the reference ("gold") standard diagnostic procedure in light of earlier findings indicating that experienced clinicians' judgment of children at the age of 18 and 24 months is a better and more stable predictor of later diagnosis than strict reliance on cut-off scores on the *ADOS*[8,9]. While *ADOS* scores for individual children may vary during the first 2 to 3 years of life, clinician best-estimate diagnosis shows more stability[9,10].

It is also worth noting that although the reference standard ASD diagnostic procedures employed in this study represent the current gold standard, they are necessarily subjective and entail some variance in clinician certainty of diagnosis. In a recent paper by other clinician-scientists[11] as well as in a multi-site study that followed the present work[12,13], expert clinicians were less than fully certain of their diagnoses in 40% and 29.5% of cases, respectively. Uncertainty in the reference standard necessarily biases the performance metrics of any comparison test downward (see eFigure 7 in Jones et al[13]).

Unfortunately, in the present study, we did not prospectively track clinician certainty of diagnosis in all children, in either the discovery study sample or the replication study sample. We expect that there were cases with less than fully certain reference standard diagnostic labels given. Some of these cases are detailed in the present document in the section titled, “Clinical Outcomes of False Positives and False Negatives”, which notes, for example, that some of the children given non-ASD reference standard labels were noted as having “subthreshold” concerns and invited to return for re-evaluation 1 year later. This information strongly suggests that these cases were in fact “uncertain diagnosis” at time of initial diagnostic evaluation. Unfortunately, however, because we did not prospectively track certainty in all participants, we were unable to analyze the effects of clinician certainty in the discovery and replication sample. This was a study design flaw that we were able to improve in the subsequent multi-site study[13].

Data Processing

Identification of Eye Movement Events

Analysis of eye movements and coding of fixation data were performed with software written in MATLAB (MathWorks). The first phase of analysis was an automated identification of non-fixation data comprising blinks, saccades and any missing data or fixations directed away from the presentation screen. Saccades were identified by eye velocity using a threshold of 30° per sec[14]. We tested the velocity threshold with the 60-Hz eye-tracking system (ISCAN, Inc) and, separately, with eye-tracking systems collecting data at 120Hz and 500Hz (SensoMotoric Instruments, GmbH); in all cases, saccades were identified reliably as compared with hand coding of the raw eye-position data. Blinks were identified as described in [15]. Missing data and off-screen fixations (when a participant looked away from the video)

were identified either by missing values in gaze vector data or by gaze vectors directed to locations beyond the bounds of the stimuli presentation monitor.

Calibration Accuracy

eFigure 4.A-D shows total variance in calibration accuracy for the discovery (A,B) and replication (C,D) study cohorts. **eFigure 4.E-H** shows average calibration accuracy. Average calibration accuracy for all groups was less than 1° of visual angle. Calibration accuracy did not differ significantly between groups. For individual variance in calibration accuracy (individual data underlying the density distributions in **eFigure 4.A-D**): in the Discovery Non-ASD sample, 93.7% of individuals had calibration accuracy within 3°, 85.1% within 2°, and 58.0% within 1°; for the Discovery ASD sample, 95.9% had calibration accuracy within 3°, 90.1% within 2°, and 66.2% within 1°; in the Replication Non-ASD sample, 96.7% of individuals had calibration accuracy within 3°, 90.0% within 2°, and 77.2% within 1°; for the Replication ASD sample, 96.2% had calibration accuracy within 3°, 95.1% within 2°, and 84.6% within 1°.

Minimum Valid Data Criterion

For each video stimulus presented (see Testing Stimuli section above for additional details), we used a minimum-valid-data criterion of fixation time greater than or equal to 20% of total recording duration, as in [4]. We set no threshold for minimum number of videos sufficient for inclusion of a child's data in analyses; if usable data were collected, with a given video stimulus fixated at a level greater than or equal to the minimum-valid criterion, then the child's data were analyzed and included. Likewise, although the goal of eye-tracking data collection was to collect data for all 14 video stimuli, children were not required to watch all stimuli for their data to be included and analyzed; if a child fell asleep during testing; cried during testing; or had a tantrum after testing had commenced, then sessions were stopped before a child completed watching all stimuli. In the discovery study, the mean number of videos watched was 5.5 ±3.8 (data given as mean ±s.d.). The maximum number of videos watched per child was 14; the mode was 4; the median was 4; and the minimum number was 1. In the replication study, of 14 possible video trials, the mean number of videos watched was 11.5 ±3.1 (data given as mean ±s.d.). The mode number of videos watched per child was 14; the median was 12; and the minimum number of videos

watched for a single participant was 1. The mean number per child of videos that failed to meet the minimum valid data criterion was 1.6 ±2.5 (data given as mean ±s.d.). The mode number of videos that failed to meet the minimum valid data criterion was 0, the median was 1, and the maximum number was 14 (leading, of course, in that case to an "index test failure" with no usable data collected, as noted in main text Figure 1; see also Supplementary Results: Missing Data section below for additional information about missing data).

Quality Control Indicators

Data processing began with calculation of a series of automated quality control indicators. The data processing software extracted information from each participant's raw data files, tested the temporal alignment of collected data, and then identified basic oculomotor events in the data (fixations, saccades, and blinks). Next, quality indicators were calculated for calibration accuracy, duration of data collected, percentage of time spent watching, number of videos watched, number of videos excluded (due to not meeting the minimum valid data criterion for that video), rates of saccades and percentage of time spent saccading, and an eye movement signal-to-noise ratio (comparing ratio of recorded eye movements to head movement artifacts). Data that failed to meet quality thresholds were excluded from further analysis. Quality thresholds were fixed values (i.e., they are non-adaptive, unchanging). Data that met or exceeded the quality thresholds proceeded to data analysis.

Additionally, eye movements identified as fixations were coded into four content-based regions of interest (ROIs) that were defined within each frame of all video stimuli as shown in **eFigure 1**: eyes, mouth, body (neck, shoulders and contours around eyes and mouth, such as hair) and objects (surrounding inanimate stimuli). The regions of interest were hand traced for all frames of each video and stored as binary bitmaps. Automated coding of fixation time to each region of interest then consisted of a numerical comparison of each infant's coordinate fixation location data with the bitmapped regions of interest.

Time-Varying Kernel Density Estimation

In order to quantify dynamic visual scanning, we used time-varying kernel density estimation[16] (**eFigure 3**). This allowed us to quantify visual scanning for groups of viewers by making nonparametric estimates of an underlying probability density function[16] for fixation and scanning data at each moment in time. Conceptually, the time-varying probability density distributions yield data-driven estimates of behavioral salience and engagement: the data demarcate areas in space and time that are most (or least) likely to be fixated by two-year-old children without ASD, and thus demarcate the timeline for out-of-sample comparisons: comparisons either with an ASD sample (as in the discovery study, to define a classification timeline), or in comparison with new children with unknown diagnoses or unknown levels of symptom severity (as in the leave-one-out cross validations in the discovery study and in the replication study).

Methodologically, this entailed 4 major steps. First, visual scanning was quantified for non-ASD normative viewers in the discovery study by kernel density estimation at each moment in time[16] (**eFigure 3A-C**). Second, moments in time were identified when the majority of discovery study non-ASD viewers fixated on the same location(s) at the same moments in time, at levels greater than expected by chance (**eFigure 3D-G**). Third, moments in time were identified when (a) the majority of discovery study non-ASD viewers fixated on the same location(s) (from step 2) but the majority of discovery study viewers with ASD fixated on alternate locations (time points used subsequently to define a classification timeline), as well as (b) time points when discovery study data were correlated with measures of social disability and cognitive functioning (timepoints used subsequently to define correlation timelines). Fourth, the performance of the classification and correlation timelines was independently tested by leave-one-out cross-validation (LOOCV) in the discovery study cohort. After completion of discovery study analyses, performance of the classification and correlation timelines were independently tested again by external validation in the replication study.

Steps 1-3 were undertaken in the discovery study. Step 2—testing whether non-ASD viewers fixated on the same location(s) at the same moments in time, at levels greater than expected by chance—was accomplished by creating simulated kernel density estimates from randomized fixation data. The simulations yielded probabilities for how frequently fixation locations—by chance—were likely

to be widely distributed or how infrequently they might converge on common locations. In this way, the simulations served as probabilistic benchmarks for the empirical data, enabling us to quantify when visual scanning of non-ASD children converged significantly (with cutoff of $p < 0.001$) (**eFigure 3G**). Step 3 yielded the test positivity threshold for diagnostic group classification, which was set as a static threshold and then applied in testing the replication study in Step 4.

Example data for time-varying kernel density estimation are shown in **eFigure 4**. In relation to the video testing stimuli (**eFigure 4A**), kernel density estimation quantifies visual scanning as a dynamic probability density function (**eFigure 4B**). Each plot in 3B is a measure of the probability of non-ASD children looking at video content at each moment in time (shown in pseudocolor, with "hotter" colors denoting greater probability, and "cooler" colors denoting lesser probability). The data can also be represented, as in **eFigure 4C**, by plotting the shape of the region most likely to be fixated over time (termed an "attentional funnel"). In the example plotted, the probability of non-ASD viewers fixating on specific onscreen content at specific moments in time becomes periodically very high, and the portion of testing stimuli fixated—the target of social visual engagement at those moments—becomes very narrowly delimited: at times of greatest convergence in 3C, the portion of video content encompassed by the attentional funnel is less than 2.03% of the image's total area (6222 of 307,200 pixels). Convergence in the attentional funnel marks instances of non-ASD normative behavior against which the behaviour of other children can be compared and quantified.

eFigure 4D shows representative plots in which the visual scanning of non-ASD children converged significantly while children with ASD fixated on alternate content (i.e., "diverged"). Each of these funnels is an example from one time point in the classification timeline. In each episode of between-group divergence in the discovery cohort data (episodes as exemplified in the plots in **eFigure 4D**), each viewer's fixation location is associated with a probability of fixating on that location at that moment in time. These probability measures, at each moment in time, are the primary quantification of behavior used to compute each of the 4 eye-tracking based indices: diagnostic classification index, social disability index, verbal ability index, and nonverbal ability index. For diagnostic classification, the optimal test-positivity threshold (i.e., the threshold used to determine "positive" for ASD [ASD] or "negative" for ASD [non-ASD]) was determined in the discovery study by calculating Youden's Index. In the discovery cohort,

performance of the prespecified threshold was calculated by leave-one-out cross-validation, determining an optimal threshold for the full discovery cohort minus one “left-out” participant, then testing threshold performance on that individual, and repeating for all individuals. The optimal threshold for the discovery study cohort as a whole was frozen and tested in the independent replication study. After determination in the discovery study, the pre-specified test-positivity threshold was set to 0 (“set to 0” by subtracting the optimal threshold at each time point from all values at that time point), so that values less than or equal to 0 indicated positive for ASD, while values greater than 0 indicated negative for ASD (non-ASD).

Data Analysis and Statistics

Sample Size and Power Considerations

The discovery and replication study sample sizes were powered with respect to two endpoints: (1) measuring sensitivity and specificity (for comparing eye-tracking index test classification relative to reference standard diagnosis), and (2) measuring correlation (for comparing eye-tracking index test severity indices relative to reference standard assessments of social disability and of verbal and nonverbal ability). Power considerations for hypothesis tests were more important to sample size in the replication study (due to data mining and algorithm development focus in the discovery study). The discovery study was powered with respect to smoothness of the probability density functions (i.e., adequate sample size to obtain smooth estimates of the computed density functions[16,17]). Desire for smooth estimates of normative non-ASD data in the discovery study led to increasing sample size in the discovery study above required minimums for power with respect to sensitivity, specificity, and correlation endpoints.

Based on preliminary data from prior studies[3,4], we believed that the eye-tracking-based index test would be capable of producing an observed sensitivity of ~80% relative to reference standard diagnosis. Therefore, in order to have a 90% chance of showing that the sensitivity of the eye-tracking-based index test was significantly greater than 70% (i.e., that the lower bound of a 95% confidence interval centered at 80% would exceed a worst case of 70%), a sample of n=180 participants with reference standard confirmed ASD was needed (one-sided alpha=0.05). Likewise, from prior studies[3,4], we believed that the eye-tracking-based index test would be capable of producing an observed specificity

of ~80% relative to reference standard diagnosis. Therefore, in order to have a 90% chance of showing that the specificity of the eye-tracking-based index test was significantly greater than 70% (i.e., that the lower bound of a 95% confidence interval centered at 80% would exceed a worst case of 70%), a sample size of n=180 participants with confirmed by reference standard as non-ASD was needed (one-sided alpha=0.05). To satisfy the sample sizes for each primary endpoint, at least 360 participants with valid results for both the eye-tracking-based index test and reference standard diagnosis were needed. For secondary endpoints, required sample sizes for adequate power in the primary endpoints exceeded those needed in secondaries to have 90% probability of detecting correlation between eye-tracking based severity indices and corresponding reference standard assessments thereof (ADOS-2 and Mullen scores).

Primary Endpoint Analyses

The primary endpoint analyses consisted of comparison of diagnostic results from the eye-tracking-based index test with the results of reference standard expert-clinician diagnosis (either 'positive' or 'negative' for ASD in each case). The comparison was tested as the proportion of reference standard clinically-positive (ASD) participants for whom the eye-tracking index test results were also positive (sensitivity; *se*), and the proportion of reference standard clinically-negative (non-ASD) participants for whom the eye-tracking index test results were also negative (specificity; *sp*). Receiver Operating Characteristic (ROC) curves and confidence intervals were plotted and their respective Area Under the Curve metrics (AUCs) calculated (as in[4]). To minimize confidence interval coverage biases, 95% confidence intervals for sensitivity and specificity were constructed for sensitivity and specificity with the Modified Wald Method[18]. False Negative Rate (FNR; defined as 1-sensitivity), False Positive Rate (FPR; defined as 1-specificty), positive predictive value (PPV), negative predictive value (NPV) and accuracy of the eye-tracking-based index test were also calculated, with corresponding 95% confidence intervals (with PPV and NPV calculated here on basis of study sample rather than population prevalence).

Secondary Endpoint Analyses

The secondary endpoint analyses consisted of measuring associations between eye-tracking-based dimensional indices and their respective expert-clinician-administered standardized reference tests (*ADOS*- and *Mullen*-based measures of social disability and of verbal and nonverbal ability). Trained expert clinicians, blind to results of the eye-tracking index test, completed all reference standard assessments. Prespecified hypotheses for testing the secondary endpoints were as follows: H_0(social): $\rho \geq -0.25$ vs. H_a(social): $\rho < -0.25$ (for correlation between ADOS-2 and the eye-tracking-based social disability index; H_0(verbal): $\rho \leq 0.20$ vs. H_a(verbal): $\rho > 0.20$ (for correlation between Mullen Verbal Age Equivalent score and the eye-tracking-based verbal ability index); and H_0(nonverbal): $\rho \leq 0.20$ vs. H_a(nonverbal): $\rho > 0.20$ (for correlation between Mullen Nonverbal Age Equivalent score and the eye-tracking-based verbal ability index).

Standard regression diagnostics[19,20] were conducted for all comparisons. Individual data points with greater than expected leverage, as measured by Cook's Distance and DFFITS (difference in fits)[20], were identified as sample outliers, using a standard metric for identifying influential observations (absolute values greater than 2 times the square root of p/N, where p is the number of regression model parameters (coefficients) and N is the comparison sample size). Individual data points with greater than expected leverage (influential observations) are denoted by ' + ' data markers in main text **Figure 3**. Pearson correlation R values and adjusted R^2 values were calculated with and without the outlier observations (**eTable 1**). Although the 95% confidence intervals of the R and adjusted R^2 estimates were overlapping in all comparisons with and without outlier observations, the magnitude of the values increased (as expected) with outliers withheld. Visual inspection of the data confirmed that the data with greater than expected leverage mark individuals with results that diverge from the bivariate population association, with scores that do not closely align with the population prediction.

For associations with ADOS-2 total scores, these discrepant measures are of interest, as they may represent cases in which (a) a child tested poorly on the ADOS but eye-tracking indices suggest potential for greater social adaptive skills than were manifest during assessment (these cases are outlier observations in the upper right corner of the scatter plot in main text **Figure 3A,3D**); or (b) a child tested well on the ADOS but eye-tracking indices suggest relatively poorer attention to social information in the

environment (these cases are outlier observations in the lower left corner of the scatter plot in main text **Figure 3A,3D**). Similarly, for associations with Mullen age equivalent scores, individual data with greater than expected leverage are most prominent in cases when a child's Mullen age equivalent scores appear higher than corresponding eye-tracking indices: this is most prominent in the upper right of main text **Figure 3F**, indicating children with high nonverbal cognitive abilities via the Mullen but relatively lower than expected corresponding eye-tracking indices. Given the expanded age-at-enrollment allowed in replication, these individuals correspond to cases with higher nonverbal cognitive age equivalents with data less well fitted by the discovery study model based on more restrictive, younger age-at-enrollment.

In **eTable 1**, we provide both Pearson correlation R values and adjusted R^2 values. Adjusted R^2 values are adjusted for test-retest reliability of the *ADOS-2* (as published in the *ADOS-2* Manual[6]) or of the *Mullen Scales of Early Learning* (as published in the *Mullen* Manual[7]). Test-retest reliability coefficients set an upper limit for which measurement variance can be accounted[21–23]. Values are reported with bootstrap 95% confidence intervals. Test-retest reliability for the reference standard instruments are 'good' but not 'excellent'[6,7]: 0.87, 0.74, and 0.75, respectively, for *ADOS-2* total score and *Mullen* verbal and nonverbal scales. These measures of test-retest reliability quantify reference standard measurement error and set an upper limit on the amount of non-error variance that can be explained by a proxy instrument[21–23].

As a related side note, when comparing two methods of measurement that measure the same attribute on the same scales with the same range, Bland-Altman methods of agreement are often desirable[24]; however, in the present case, we have two methods of measurement that measure common constructs (e.g., "social disability", "verbal ability") but do so by measuring different attributes with different measurement scales and different ranges. Bland-Altman analyses are not appropriate to comparison of measures with different scales and ranges. In the current analyses, our focus was not on absolute (Bland-Altman-style) agreement, but on concurrent and convergent validity. Deming regression[25,26], together with Pearson correlation R values, and adjusted R^2 values were used to assess the relationships between the eye-tracking-based Social Disability, Verbal Ability and Nonverbal Ability indices and the *ADOS* Total score, *Mullen* verbal and nonverbal age equivalent scores, respectively.

Data Sharing Statement

The data that support the findings of this study will be publicly reposited in the NIMH Data Archive (https://nda.nih.gov), with accession number added when available.

© 2023 Jones W et al. *JAMA Network Open*.

eResults. Clinical Outcomes of False Positives and Negatives and Missing Data

Clinical Outcomes of False Positives and Negatives

In the replication study, there were 32 false positives (participants for whom the eye-tracking-based index test indicated ASD while the reference standard clinical diagnosis indicated non-ASD). 20/32(62.5%) were flagged for clinical concern. Amongst these 20, 80% (16), although not diagnosed with ASD, were instead diagnosed with a different developmental disorder or delay, or were given provisional/subthreshold diagnoses of social communication disorders (and asked to return for additional testing in one year). Ten of these false positive children were given labels of either “Subthreshold ASD” (7 children) or “Subthreshold Communication Disorder” (3 children). These labels are often used by clinicians adopting a “wait-and-see” approach, which typically reflects a concern that the child may in fact have ASD but the child’s current presentation is not believed to be obvious enough yet to warrant a formal ASD diagnosis. Five additional children were given diagnoses of either “Communication Disorder” (3 children) or “Cognitive/Developmental Delay” (2 children), and 1 child was referred for genetic testing due to dysmorphology (given a reference standard label of “Suspected Genetic Condition”). Amongst the 12 who were not flagged for clinical concerns, 6 were noted as having somewhat delayed speech understanding or production (via percentile scores on the *MacArthur-Bates Communicative Development Inventories (CDI*), with 3 scoring in the lowest population decile and 3 in the lowest quartile for words understood or words produced).

It is worth noting that for the 16 false positives who were diagnosed with different developmental disorders or given provisional/subthreshold diagnoses, these children—while technically false positives for ASD in comparison with the reference standard expert clinician diagnosis—are also children who needed and can benefit from early identification. For children diagnosed with other intellectual and developmental disabilities, early identification with an ASD diagnosis would nonetheless allow those children to receive early intervention needed to support their developmental delays, given that social communication therapies used for children with ASD also promote optimal outcomes in children with other speech-language-communication delays and intellectual and developmental disabilities.

In the replication study, there were 35 false negatives (participants for whom the eye-tracking-based index test indicated non-ASD while the reference standard clinical diagnosis indicated ASD). The

false negatives presented as having significantly higher Mullen scores ($t = 3.71$, $p < 0.001$) as well as significantly lower ADOS scores ($t = 5.17$, $p < 0.001$) than true positives.

For both false positives and false negatives, it will be of interest to examine individual case profiles in greater detail, and also, for the sample as a whole, to endeavor to collect later age follow-up data to learn more about the eventual outcomes and developmental trajectories of children tested here at approximately 2 years of age.

Missing Data

Data processing and analysis for the eye-tracking-based index test were fully automated and included a series of quality control indicators. If eye-tracking data did not meet or exceed pre-set, static quality control thresholds determined on the basis of prior published work[3,4,27–29], then further analysis was not performed. As shown in main text **Figure 1** (recorded in the study flow diagram as “index test failures”), in the discovery study, eye-tracking data from 8 participants failed to meet the quality control thresholds; in the replication study, eye-tracking data from 9 participants failed to meet the quality control thresholds.

The quality control indicators included measures of calibration accuracy, duration of data collected, percentage of time spent watching, number of videos watched, number of videos excluded, and percentage and rates of saccades. Data that failed to meet all quality control thresholds were excluded from analysis; only data that met or exceeded quality control thresholds were analyzed. As noted above in the “Inclusion and Exclusion Criteria” section, discovery study participation was restricted to children who successfully completed an entire lab-based eye-tracking data collection session and could therefore provide high quality eye-tracking data to algorithm development; by design, this means that the data collection failure rate in the discovery study (8/719 or 1.1% who completed a full testing session but then failed quality control indicators) is likely to be optimistically biased. No such restrictions were placed on the replication study (9/370 or 2.4%).

eTables 2 and **3** list the reasons why data for 8 children in the discovery study and 9 children in the replication study failed to meet criteria for valid data inclusion. All checks were automated with static, pre-set thresholds. The eye movement signal-to-noise ratio quality control indicator (QCI) and the

saccade percentage and rate QCI were further developed in the discovery study for use in replication, and so were not used as QCIs in the discovery study. After unblinding in both studies, it was determined that among children with insufficient eye-tracking data for analysis, their reference standard diagnoses were as follows (as also shown in main text **Figure 1**): in the discovery study, 7 had ASD and 1 did not, and in the replication study, 6 had ASD and 3 did not.

We would note that these are data collected from children approximately 2 years old; that the majority of these children were either non-verbal or minimally verbal; that 519 of these children had a developmental disability (autism) which directly affects their ability to follow and respond to social communication (e.g., verbal instructions); and that behavioral challenges and outbursts (crying, tantrums, kicking, or general non-compliance) are all very commonly observed in children both with and without autism in this age range.

The high rate of successful data collection (361/370 in the replication study) of objective, quantitative, and performance-based measures of social functioning is a strong feature of the index test. The eye-tracking index test—in semi-automated fashion, operated by a non-specialist technician—collected measurements that can proxy those of expert clinicians, and successfully collected data to do so in 97.6% of children.

It is also worth considering missing data relative to worst case and best case scenarios regarding how missing results might have impacted sensitivity and specificity. In a worst case scenario (i.e., treating every child with missing eye-tracking data [those who failed the quality control indicators] as either a false positive or a false negative, then (as would be expected) sensitivity and specificity are both reduced slightly, to se = 78.0% (95% CI: 71.4%-83.3%) and sp = 81.0% (95% CI: 74.7%-86.0%), but the reduction is not significantly different from the actual results. Likewise, in a best case scenario (i.e., treating every child with missing eye-tracking data as either a true positive or a true negative, then (as would be expected) sensitivity and specificity are both increased slightly, to se = 81.2% (95% CI: 74.9%-86.2%) and sp = 82.6% (95% CI: 76.4%-87.4%). Again, the increase is not significantly different from the actual results. Thus, under either best case or worst case scenarios for missing data, the test sensitivity and specificity do not change significantly.

eReferences

1. United States Food and Drug Administration. *Statistical Guidance on Reporting Results from Studies Evaluating Diagnostic Tests - Guidance for Industry and FDA Staff*.; 2007.

2. Lijmer JG, Mol BW, Heisterkamp S, et al. Empirical evidence of design-related bias in studies of diagnostic tests. *JAMA*. 1999;282(11):1061-1066.

3. Jones W, Klin A. Attention to eyes is present but in decline in 2-6-month-old infants later diagnosed with autism. *Nature*. 2013;504(7480):427-431. doi:10.1038/nature12715.

4. Constantino JN, Kennon-McGill S, Weichselbaum C, et al. Infant viewing of social scenes is under genetic control and is atypical in autism. *Nature*. 2017;547(7663):340-344. doi:10.1038/nature22999.

5. Leckman JF, Sholomskas D, Thompson D, Belanger A, Weissman MM. Best Estimate of Lifetime Psychiatric Diagnosis: A Methodological Study. *Arch Gen Psychiatry*. 1982;39(8):879-883. doi:10.1001/archpsyc.1982.04290080001001.

6. Lord C, Rutter M, DiLavore P, Risi S, Gotham K, Bishop S. *Autism Diagnostic Observation Schedule, Second Edition: ADOS-2*. Torrance, CA: Western Psychological Services; 2012.

7. Mullen EM. *Mullen Scales of Early Learning*. AGS. Circle Pines, MN, USA: American Guidance Service; 1995.

8. Kim SH, Lord C. Combining information from multiple sources for the diagnosis of autism spectrum disorders for toddlers and young preschoolers from 12 to 47 months of age. *J Child Psychol Psychiatry Allied Discip*. 2012;53(2):143-151. doi:10.1111/j.1469-7610.2011.02458.x.

9. Chawarska K, Klin A, Paul R, Volkmar F. Autism spectrum disorder in the second year: Stability and change in syndrome expression. *J Child Psychol Psychiatry*. 2007;48(2):128-138. doi:10.1111/j.1469-7610.2006.01685.x.

10. Chawarska K, Klin A, Paul R, Macari S, Volkmar F. A prospective study of toddlers with ASD: short-term diagnostic and cognitive outcomes. *J Child Psychol Psychiatry*. 2009;50(10):1235-1245. doi:10.1111/j.1469-7610.2009.02101.x.

11. McDonnell CGG, Bradley CCC, Kanne SMM, Lajonchere C, Warren Z, Carpenter LAA. When Are We Sure? Predictors of Clinician Certainty in the Diagnosis of Autism Spectrum Disorder. *J Autism Dev Disord*. 2019;49(4):1391–1401.

12. Klaiman, C., White, S., Richardson, S., McQueen, E., Walum, H., Aoki, C., Smith, C., Minjarez, M., Bernier, R., Pedapati, E., Bishop, S., Ence, W., Wainer, A., Moriuchi, J., Tay, S. W., Deng, Y., Jones, W., Gillespie, S., & Klin A. Expert Clinician Certainty in Diagnosing Autism Spectrum Disorder in 16-30-Month-Olds: A Multi-site Trial Secondary Analysis. *J Autism Dev Disord*. 2022:1-16.

13. Jones W, Klaiman C, Richardson S, et al. Eye-Tracking-Based Measurement of Social Visual Engagement Compared with Expert Clinical Diagnosis of Autism: A Multi-Site Study. *Under Rev*.

    Please note that reference 13 is currently under review at JAMA and will be updated at the time of publication.

14. Leigh RJ, Zee DS. *The Neurology of Eye Movements*. 4th ed. Oxford University Press, USA; 2006.

15. Shultz S, Klin A, Jones W. Inhibition of eye blinking reveals subjective perceptions of stimulus

salience. *Proc Natl Acad Sci U S A*. 2011;108(52):21270-21275. doi:10.1073/pnas.1109304108.

16. Silverman BW. *Density Estimation for Statistics and Data Analysis*. London: Chapman and Hall; 1986.

17. Shimazaki H, Shinomoto S. Kernel bandwidth optimization in spike rate estimation. *J Comput Neurosci*. 2010;29(1-2):171-182. doi:10.1007/s10827-009-0180-4.

18. Agresti A, Coull B. Approximate is better than “exact” for interval estimation of binomial proportions. *Am Stat*. 1998;52(2):119-126.

19. Bollen KA, Jackman RW. Regression diagnostics: An expository treatment of outliers and influential cases. *Sociol Methods Res*. 1985;13(4):510-542. doi:10.1177/0049124185013004004.

20. Kutner M, Nachtsheim C, Neter J, Li W. *Applied Statistical Linear Models*. 5th ed. New York, NY: McGraw-Hill; 2005.

21. Spearman C. The proof and measurement of association between two things. *Am J Psychol*. 1904;15(1):72-101.

22. Law MR, Wald NJ, Wu T, Hackshaw A, Bailey A. Systematic underestimation of association between serum cholesterol concentration and ischaemic heart disease in observational studies: data from the BUPA study. *BMJ*. 1994;308(6925):363-366.

23. Charles EP. The correction for attenuation due to measurement error: clarifying concepts and creating confidence sets. *Psychol Methods*. 2005;10(2):206-226.

24. Bland JM, Altman DG. Statistical methods for assessing agreement between two methods of clinical measurement. *Lancet*. 1986;1(8476):307-310.

25. Linnet K. Performance of Deming regression analysis in case of misspecified analytical error ratio in method comparison studies. *Clin Chem*. 1998;44(5):1024-1031.

26. Martin RF. General deming regression for estimating systematic bias and its confidence interval in method-comparison studies. *Clin Chem*. 2000;46(1):100-104.

27. Jones W, Carr K, Klin A. Absence of preferential looking to the eyes of approaching adults predicts level of social disability in 2-year-old toddlers with autism spectrum disorder. *Arch Gen Psychiatry*. 2008;65(8):946-954. doi:10.1001/archpsyc.65.8.946.

28. Klin A, Lin DJ, Gorrindo P, Ramsay G, Jones W. Two-year-olds with autism orient to non-social contingencies rather than biological motion. *Nature*. 2009;459(7244):257-261. doi:10.1038/nature07868.

29. Moriuchi JM, Klin A, Jones W. Mechanisms of diminished attention to eyes in Autism. *Am J Psychiatry*. 2017;174(1). doi:10.1176/appi.ajp.2016.15091222.
