# Supplementary material for: Development and Replication of Objective Measurements of Social Visual Engagement to Aid in Early Diagnosis and Assessment of Autism
Source: JAMA Netw Open. 2023 Sep 5;6(9):e2330145. doi: 10.1001/jamanetworkopen.2023.30145 (PMC10481232; doi:10.1001/jamanetworkopen.2023.30145)
Supplement: Supplement 2. — Data Sharing Statement [file jamanetwopen-e2330145-s002.pdf]

Data Sharing Statement

Jones. Development and Replication of Objective Measurements of Social Visual Engagement to Aid in Early Diagnosis and Assessment of Autism. *JAMA Netw Open*. Published September 05, 2023. doi:10.1001/jamanetworkopen.2023.30145

Data
Data available: Yes
Data types: Deidentified participant data, Data dictionary
How to access data: The data that support the findings of this study will be publicly reposited in the NIMH Data Archive (https://nda.nih.gov), with accession number added when available.
When available: With publication

Supporting Documents
Document types: None

Additional Information
Who can access the data: Data availability will follow standard NIH NDA policies, as described at https://nda.nih.gov/faq.html#dac.1
Types of analyses: Data analysis purposes of shared data will also follow standard NIH NDA policies, as described at https://nda.nih.gov/faq.html#top
Mechanisms of data availability: NIH NDA data use requires a signed data use certification, described at https://nda.nih.gov/faq.html#dac.3
